# Measuring extremist archetypes: Scale development and validation

**DOI:** 10.1371/journal.pone.0270225

**Published:** 2022-07-20

**Authors:** Milan Obaidi, Sara W. Skaar, Simon Ozer, Jonas R. Kunst

**Affiliations:** 1 Department of Psychology, University of Oslo, Oslo, Norway; 2 Center for Research of Extremism, University of Oslo, Oslo, Norway; 3 Department of Psychology, Aarhus University, Aarhus, Denmark; University of Padova, ITALY

## Abstract

Previous work has often disregarded the psychological heterogeneity of violent extremists. This research aimed to contribute to a more nuanced understanding of the psychological diversity of violent extremists. Based on qualitative work, we developed and validated the Extremist Archetypes Scale, identifying five distinct archetype dimensions: “adventurer,” “fellow traveler,” “leader,” “drifter” and “misfit.” Study 1 identified five dimensions among White majority members (*N* = 307), four of which were related to extremist violent intentions and which dissociated in terms of sociopolitical ideologies and intergroup attitudes. Preregistered Study 2 (*N* = 308) confirmed the scale’s five-factor solution in another sample of White majority members, replicated relationships with violent intentions, and demonstrated the dimensions’ distinct personality correlates. As in Study 1, the archetype dimensions had positive associations with extremist violent intentions and tapped onto different psychological profiles in terms of major personality traits. Study 3 (*N* = 317) replicated these results in a sample of Muslim minority members. Measurement equivalence was established across gender, age, political orientation, and ethnicity (majority and minority).

## Measuring extremist archetypes: Scale development and validation

In a review of the literature [1] concluded that most studies and theories of violent extremism fail to take into account that violent extremists are a highly heterogeneous group, especially with regard to temperament, ideology, and cognitive capacities. Although extremist organizations usually expect all group members to strive for common goals, in reality, there is often significant variation across individual members’ motivations, behaviors, personalities, preferences, and knowledge. As such, there may be limited grounds for approaching the question of who becomes involved in violent extremism, even within a single specific group, in a homogeneous fashion [2]. Nevertheless, much previous research has disregarded the motivational and social heterogeneity of violent extremists that has been observed in qualitative work [e.g., 3,4]. To address this gap, we set out to develop a scale of “extremist archetypes,” reflecting specific patterns of behavior, thoughts, and personality traits.

Based on existing qualitative typologies, we tested whether extremist archetypes can be operationalized and validated at a quantitative level. Validation of such a typology and the construction of a quantitative measure to reflect it would be an important stepping stone to further empirical insights into the heterogeneity that characterizes violent extremists [1]. Although repeatedly acknowledged as a limitation of violent extremism research, validated scales to assess the heterogeneity of violent extremists are still lacking [2].

### The potential role of archetype dimensions

Qualitative research has provided valuable and often neglected insights into how individual-level differences are connected to people’s involvement in violent extremism. Bjørgo [3] identified four extremist archetypes among right-wing extremists that are characterized by different backgrounds, motivations, psychological profiles, and roles within an extremist movement. First, the “ideological activist” is thought to be primarily driven by political and ideological motives and is typically resourceful and idealistic. They often occupy leadership roles, are less inclined to use violence themselves, and may play an important role in radicalizing others. The existence of this archetype is supported by a recent empirical study demonstrating that even though leaders are more ideologically committed to the group’s goals and ideology, they are at the same time less likely to engage in violent acts [5]. The “fellow traveler” extremist is driven by a need for belonging, friendship, and acceptance. This archetype is thought to gradually become radicalized as a consequence of being part of a group. Indeed, in the literature on violent extremism, the need for belonging and identity have been persistently identified as key factors in the process of radicalization [6,7]. Bjørgo [3] termed the third archetype “socially frustrated,” marking a person who is not ideologically oriented but struggles socially and harbors negative emotions such as anger and aggression that can be channeled into targeting an enemy. Violent extremists of this type often have a challenging social background and find roles in a militant group in which they may receive recognition for their violent and criminal experience. This aligns with theoretical accounts and empirical studies demonstrating that a quest for meaning and the need for recognition and social approval are potent factors that motivate people to engage in violent extremism [8]. The last extremist type in Bjørgo’s [3] taxonomy is termed the “adventurer,” or one who is thought to be involved in violent extremism primarily because of the excitement, action, and opportunity to be a heroic fighter. Indeed, in support of this archetype, previous research found a positive correlation between a desire to seek out situations marked by danger and excitement and willingness to participate in suicide terrorism, politically motivated organized violence, and homegrown terrorism in the West [9].

In an dependently study, Nesser [4] also proposed four different archetypes based on European jihadists’ personal accounts before, during, and after their involvement in terrorist activities [3]. Nesser’s classification only partly deviates from the typology Bjørgo proposed. First, the “entrepreneur,” similar to Bjørgo’s “leader,” plays an important role in the planning of a terrorist attack and is often smart, well-educated, and resourceful, as well as driven by religious and political convictions. Second, the “protégé” often acts as the right hand of the entrepreneur and plays an important role in recruitment. Third, the “misfit,” similar to Bjørgo’s “socially frustrated” extremist, has had a difficult childhood and feels unjustly victimized. Next, the “misfit” shows a greater willingness to make personal sacrifices and therefore may be willing to commit an actual violent attack. Finally, the “drifter” is primarily driven by a need for group belonging, recognition, and acceptance rather than religious or political convictions.

As stated earlier, the above descriptions tap into both the social roles and psychological profiles of different extremist archetypes. Researchers argue that individual psychological factors such as nonclinical personality traits can be useful in advancing our understanding of violent extremism [10–12]. For instance, recent research has established an association between basic personality characteristics and more extreme ideological standpoints [12]. Others have found a pattern of personality traits expressed through low intellect/imagination, low extraversion, and high agreeableness that seem to make people vulnerable to extremist ideology. In addition, recent research indicated that 11–27% of the individual differences in violent tendencies can be explained by basic personality traits [e.g., low openness and low emotionality; 12]. In relation to the Dark Triad personality inventory, a recent empirical study demonstrated a link between the Dark Triad inventory and violent intentions [13]. Taken together, given that personality traits play a role in violent extremism, they are also likely to underpin extremist archetype dimensions.

To validate our scale, we aimed to test the extremist archetypes’ associations with HEXACO-PI-R inventory [14] and Dark Triad personality traits [15]. In addition, we aimed to validate the scale in terms of sociopolitical attitudes, ideologies, prejudice, and ethnic identification, as these constructs commonly play a role in political extremism and out-group intolerance [16,17]. Finally, as recommended by Hussey and Hughes [18], we sought to establish measurement equivalence across gender, political orientation, and majority-minority group status to develop a scale that is applicable in diverse settings.

As our work is exploratory, we were generally agnostic in our predictions regarding how personality would relate to each archetype. Nevertheless, one may have expected certain associations. First, a positive association between the “adventurer” and extraversion and openness to experience may be plausible because people who score higher on these traits are often marked in turn by inquisitiveness, creativity, and unconventionality (e.g., openness), and they are typically outgoing and excitement seeking (extraversion). The adventurer archetype is described as excitement- and action-oriented and novelty-seeking.

The emotionality dimension represents the tendency to experience a range of emotional reactions, including fear, anxiety, need for emotional support, and empathy. Therefore, one may expect an association between emotionality and the “fellow traveler” dimension that describes people who join militant group activities due to their need for acceptance and recognition.

Honesty-humility encompasses traits of sincerity, fairness, greed avoidance, modesty, and reluctance to exploit others [19]. People high on honesty-humility are less likely to engage in transgressive and norm-violating behavior such as petty crime [20,21]. The agreeableness dimension characterizes the tendency to be pleasant, tolerant, helpful, trusting, forgiving, considerate, and cooperative. Previous work has established a negative relationship between agreeableness and deviant behavior [22]. The conscientiousness dimension is described by traits such as trustworthiness, competence, achievement striving, self-discipline, and dutifulness, and previous studies found a negative relationship between conscientiousness and a variety of behaviors that are deviant and harmful [23]. Therefore, it may be reasonable to expect a negative association between honesty-humility, agreeableness, conscientiousness, and the “misfit” archetype who is more likely to engage in a variety of deviant behaviors such as petty crime and hostility toward others. Similarly, we will likely observe a negative association between honesty-humility, agreeableness, and conscientiousness and the “drifter” archetype as the archetype is described as impulsive and unconcerned with routines and order and as driven mainly by personal gain, need for belonging recognition, and acceptance. Finally, leadership success has been known to correlate with openness, extraversion, and conscientiousness [24]. Hence, based on previous research, one may expect a positive relationship between extraversion, conscientiousness, and openness and the “leader” archetype.

We note that previous research focusing on personality and violent extremism has been criticized for (a) not using well-validated personality inventories to measure personality [25], (b) failing to report the methods by which personality was assessed [e.g., 26,27], and/or (c) relying on ad hoc trait measures [e.g., 28]. Other scholars have also noted that there is lack of studies linking violent extremism and personality using standard personality tests [29]. To address these shortcomings, we employed the HEXACO-PI-R inventory to provide a comprehensive mapping of human personality as it has proven to be a valid instrument across cultures [30]. The HEXACO model was initially introduced to account for observations that in many languages there is evidence of six broad personality dimensions [e.g., honesty-humility, 31]. Our interest in the HEXACO model was also guided by recent research suggesting that the last extracted factor, honesty-humility (feeling little temptation to break rules or take advantage of others), is an important predictor of hostile group attitudes [e.g., 32].

### The present research

The main objective of the present research was to construct and validate “The Extremist Archetypes Scale” in three studies. In Study 1, we piloted a larger item pool and used factor analyses to develop a multidimensional scale in a sample of White majority-group members. Further, this first study investigated the scale’s criterion and convergent validity by testing its relationship with violent behavioral intentions, sociopolitical ideologies, intergroup attitudes, and ethnic identification. In the preregistered Study 2, we aimed to confirm the scale’s factor structure and again validate it—this time in terms of associations with HEXACO and Dark Triad personality traits in addition to violent behavioral intentions using another sample of White majority-group members. In Study 3, we aimed to replicate the results of Study 2 in a sample of Muslim minority-group members. Finally, we tested whether the instrument showed measurement invariance (i.e., equal factor structure, loadings, intercepts) across gender, age, political orientation, and ethnicity/group status (i.e., majority and minority).

Acknowledging that Bjørgo [3] and Nesser [4] created taxonomies in which violent extremist individuals are assigned to one specific archetype, we for several reasons conceptualized the archetypes as continuous dimensions. From a statistical perspective, using continuous archetype scores provides more flexibility and limits the minimization of data. That is, in many cases, people may not clearly fall within one single archetype, and a dimensional perspective better captures these nuances. This is especially important because we aimed to develop a scale that can be used not only among extremist individuals but also among the general population to capture people’s gradual transition into certain archetypes.

## Study 1

Based on Bjørgo’s [3] and Nesser’s [4] qualitative insights, an item pool intended to operationalize extremist archetype dimensions was administered to a sample of White Americans. Next, participants completed a violent behavioral intentions measure [17] as well as measures that are typically associated with out-group negativity or even hostility, such as social dominance orientation [SDO; 33], right-wing authoritarianism [RWA; 34], nationalism [35], ethnic intolerance, and ethnic identification [36]. This allowed us to investigate whether the different archetypes were related to extremist tendencies, supporting the scale’s predictive (e.g., violence) and criterion/convergent (e.g., SDO, RWA, nationalism) validity.

### Method

#### Participants

In Exploratory Factor Analyses (EFA), it is recommended that the ratio of participants to variables is set to 5:1 [37], which in our analysis would be 255 participants. Hence, we aimed to recruit a sample above this minimum. We sampled in total 307 White/Caucasian Americans (*M*_age_ = 41.26, *SD*_age_ = 11.91, range = 22–71, 51.5% female). The sample was relatively heterogeneous regarding political orientation, where nearly half of the participants identified as Democrats (43.6%), and the remaining identified as Republicans (24.4%) or Independents (28.7%). A comprehensive overview of demographic variables can be found in the supplementary online material (SOM).

#### Procedure

Data were collected in October 2019 through Amazon Mechanical Turk, and the participants were paid the equivalent of 6–7 USD/hour. The study was approved by the ethical review board of Oslo University, department of psychology. An informed, written consent was obtained from the participants before participation and it was recorded electronically. Only participants 18 years of age or older were eligible to participate in this study. The data for Study 1 and the remaining studies (e.g., Studies 2 and 3) could be found at https://osf.io/nm8da.

#### Instruments

Unless stated otherwise, responses were rated on 7-point Likert scales, ranging from 1 (*strongly disagree*) to 7 (*strongly agree*). All items can be found in the SOM.

*Preliminary item pool of the extremist archetypes scale*. To establish a preliminary item pool, we used the typologies developed by Bjørgo [3] and Nesser [4] as bases. As described earlier, Bjørgo and Nesser each distinguish four main types of extremists. Due to some overlap between the two typologies and descriptions, we based the items on five possible extremist archetypes: 1) the *“*ideological activist”/“entrepreneur” (a leader type), 2) the “fellow traveler,” 3) the “drifter,” 4) the “misfit”/“socially frustrated,” and 5) the “adventurer.” We chose not to assess the “protégé” from Nesser’s taxonomy because it substantially overlaps with Bjørgo’s leader type.

Building on the existing literature and descriptions of these five archetypes, we developed a pool of 51 items with 10 to 11 items in each proposed archetype category (see SOM for all items). The items were randomized in the survey and introduced with the following text:

“Most people have some opinions regarding political issues and often support specific political movements. We would like you to think of the political group or movement you belong to or feel close to when answering the following questions. Please indicate to what extent you agree with the various statements regarding yourself.”

*Social dominance orientation*. The degree of participants’ SDO was measured using the short-form 8-item SDO_7_ scale [38, α = .94].

*Right-wing authoritarianism*. The 15-item version of the RWA scale [39] was used (α = .95).

*Nationalism*. To measure nationalism, we adapted the four-item nationalism scale by Weiss [36] to the American context (α = .79).

*Ethnic intolerance*. A four-item version of the ethnic intolerance scale was used [36, α = .86].

*Ethnic identification*. The three-item self-categorization scale by [40] was used to measure ethnic identification (α = .87).

*Violent intentions*. To measure violent intentions, we used a seven-item scale on violent behavioral intentions [17, α = .92]. We included the Violent Behavioral Intentions scale as a dependent variable as this scale has recently been validated with regard to actual radicalized behavior [see 12,41].

#### Analysis of data

First, an EFA of all 51 items using maximum likelihood was conducted. Based on the results, for each archetype dimension, the six items with the strongest factor loadings were retained and used to compute mean-scored scales. Second, to gather information about the scale’s validity, correlation analyses were conducted to test whether the subscales were associated with the validation measures as expected. Anticipating some of the archetype subscales to correlate, we estimated both bivariate and partial correlations to obtain information about the robustness of the statistical relationships. Moreover, to test whether the archetype subscales would predict participants’ violent intentions even when controlling for alternative predictors (e.g., SDO, RWA, ethnic intolerance), hierarchical multiple regression analyses were conducted.

### Results

#### Exploratory factor analysis

Kaiser-Meyer-Oklin statistics were satisfactory (KMO = .94). Bartlett’s test of sphericity was significant at *p* < .001, and correlations between items were sufficiently large (+/- .32) and did not show singularity (i.e., not stronger than +/- .90). Based on eigenvalues (Kaiser criterion), EFA yielded an eight-factor solution. As Kaiser’s criterion often overestimates the number of factors when introducing more than 30 items [42], we sought a more reliable result by using a parallel analysis that compared the eigenvalues in our sample with expected eigenvalues occurring at random. According to the parallel analysis, a five-factor solution was supported and retained. The five factors accounted for 56.52% of the total variance (see Table 1). Direct oblimin rotation provided the most parsimonious factor loadings. Of the total pool of items, 41 could be identified that substantially (>.39) loaded on the same factor with no cross-loadings above .32. Factor 5 (the “misfit”) had the lowest number of items, with a total of six meeting these criteria. Therefore, for each factor, we retained the items with the highest factor loadings (see Table 1), ending up with 30 items in total. The full pool of items can be found in the SOM. Cronbach’s alpha values were acceptable to excellent for each subscale (see Table 1).

**Table 1:** Factor loadings for exploratory factor analysis with direct oblimin rotation.

Item	Adventurer	Traveler	Drifter	Leader	Misfit
46. I am known as a group member that is not afraid to take any risks.	**.88**	-.05	-.03	.05	.08
45. I am known as a group member that is not afraid of facing dangers.	**.85**	-.07	-.01	.05	.09
44. I am willing to take more risks than other people in my group.	**.81**	-.13	-.09	.03	-.07
48. Other group members see me as someone who likes thrills and adventure.	**.80**	-.05	.02	.03	.02
43. I like groups that bring along adventure and adrenaline.	**.79**	.11	-.08	-.06	.06
49. I like to show other group members that I am not afraid of taking risks.	**.73**	-.02	-.03	.12	-.14
15. I try to find a group that accepts me.	-.12	**.64**	-.16	.08	.02
19. To belong to a group, I am willing to do what is asked of me.	.24	**.55**	.19	.04	-.11
13. I tend to "go with the flow" in group settings.	-.01	**.52**	-.07	-.29	.00
40. The group I belong to gives me stability in life.	.03	**.50**	.19	.26	-.24
25. I often join groups that others have introduced me to.	.02	**.42**	-.09	.21	-.18
24. I stay in a group as long as they give me what I need.	-.11	**.41**	-.20	.17	-.12
27. Sometimes, I suddenly decide to leave a group and seek a new one.	.11	.03	**-.66**	-.02	.05
35. Throughout my life I have found it difficult to find a group to belong to.	-.01	.02	**-.61**	-.16	.03
28. It has happened that I just replaced one group I belonged to with another.	.03	.04	**-.59**	.13	-.10
23. I rarely stay with one group for a long period of time.	.04	-.18	**-.59**	-.02	-.08
22. I have shifted a lot between groups in my life.	.05	.13	**-.59**	.16	-.04
47. I get bored more easily than other group members.	.02	-.16	**-.48**	.04	-.15
11. Most group members see me as a leader.	.15	-.01	-.03	**.82**	.05
1. In a group, I often take the role of a leader.	.15	-.07	.01	**.82**	.00
5. Other group members turn to me for guidance.	.15	.24	-.03	**.66**	.13
4. I often do most of the strategic planning in the group I belong to.	.15	.18	.02	**.65**	.05
8. I take a central position in the group I belong to.	.12	.11	-.01	**.63**	-.11
6. Other group members usually do as I say.	.14	.12	.03	**.62**	-.09
12. I often "blindly" follow my group.	-.06	.05	-.17	-.18	**-.67**
16. I am willing to change my beliefs for the group I belong to.	.11	.02	.07	-.01	**-.63**
14. I am willing to do whatever it takes to get my group to accept me.	.06	.12	.02	.17	**-.59**
33. I care little about people outside my group.	.11	-.24	-.25	.01	**-.52**
17. I put the group first and myself second.	.12	.23	.22	-.01	**-.45**
41. Before I became part of my group, I had no clear direction in life.	.14	.09	-.23	-.14	**-.45**
Eigenvalues	16.15	4.84	3.98	2.17	1.69
% of variance explained	31.67	9.49	7.80	4.25	3.31
Cronbach’s alpha	.93	.74	.78	.91	.78

*Note*. Factor loadings > .40 are boldface. Traveler = Fellow Traveler.

#### Predictive validity

We expected the different extremist archetypes to be associated with higher levels of violent intentions, which would support the scale’s predictive validity. Weak to moderate zero-order correlations between violent behavioral intentions and the “adventurer,” the “drifter,” the “misfit,” and the “leader” archetypes were found (see Table 2 for bivariate correlations). “Fellow traveler” was the only archetype not correlated with violent behavioral intentions.

**Table 2:** Correlations between the scales.

Scale	1.	2.	3.	4.	5.	6.	7.	8.	9.	10.
1. Adventurer										
2. Fellow Traveler	.22***									
3. Leader	.64***	.32***								
4. Drifter	.33	.11	.16**							
5. Misfit	.44***	.47***	.30***	.33***						
6. Social Dominance Orientation	.15*	-.08	.16**	.20***	.16**					
7. Right-Wing Authoritarianism	.15**	.09	.14*	.10	.27***	.51***				
8. Nationalism	.28***	.21***	.28***	.25***	.31***	.50***	.69***			
9. Ethnic Intolerance	.28***	.01	.15**	.23***	.35***	.59***	.55***	.50***		
10. Ethnic Identification	.13*	.40***	.20***	-.001	.28***	.27***	.46***	.53***	.35***	
11. Violent Behavioral Intentions	.43***	.01	.23***	.30***	.25***	.40***	.30***	.37***	.47***	.13*

*Note*. *p < .05

**p < .01

***p < .001.

In more conservative partial correlation analyses, both the “adventurer” and “drifter” still had positive correlations with violent behavioral intentions when controlling for the other archetypes (see Table 3). The archetype “fellow traveler” had an unexpected negative partial correlation with violent behavioral intentions, possibly suggesting a suppressor effect.

**Table 3:** Psychometric properties and partial correlations of the scale.

					Partial correlations									
					Adventurer		Traveler		Leader		Drifter		Misfit	
Scale	Items	*M*	SD	Skew	*r*_p_	*p*	*r*_p_	*p*	*r*_p_	*p*	*r*_p_	*p*	*r*_p_	*p*
Extremist Archetypes														
Adventurer	6	3.02	1.42	.32										
Fellow Traveler	6	4.37	1.01	-.82	-.12	.039								
Leader	6	3.46	1.40	.19	.60	< .001	.24	< .001						
Drifter	6	3.02	1.07	.21	.21	< .001	-.04	.511	-.06	.271				
Misfit	6	2.61	1.03	.58	.30	< .001	.43	< .001	-.06	.311	.22	< .001		
Ideology														
Social Dominance Orientation	8	2.46	1.58	.88	-.04	.484	-.20	< .001	.15	.011	.15	.010	.15	.008
Right-Wing Authoritarianism	15	2.92	1.39	.38	-.01	.869	-.06	.284	.06	.270	.01	.933	.22	< .001
Nationalism	4	3.58	1.34	-.23	.03	.561	.06	.326	.13	.026	.15	.012	.14	.016
Ethnic Intolerance	4	2.27	1.34	1.07	.11	.062	-.18	.002	.00	.969	.09	.134	.29	< .001
Ethnic Identification	3	4.65	1.30	-.55	-.03	.575	.29	< .001	.08	.184	-.09	.129	.13	.025
Violence														
Violent Behavioral Intentions	7	2.41	1.35	.92	.30	< .001	-.13	.026	-.02	.695	.17	.004	.09	.120

*Note*. Potential range for the scales = 1–7. *r*_*p*_ = partial correlations. Control variables in the partial correlations are the remaining archetypes.

To test whether the different extremist archetypes predicted violent intentions, over and above ideology, prejudice, and ethnic identification, hierarchical regression analyses with two steps were conducted. In Step 1, SDO, RWA, ethnic intolerance, ethnic identification, and nationalism were entered; in Step 2, the archetypes were added to the model. By adding the extremist archetypes, the regression model explained 11% more of the variance (total variance = 34%) in violent intentions, *F*_Change_(5, 297) = 9.74, *p* < .001. In this model, of the archetype dimensions, the “adventurer” and the “drifter” significantly predicted more violent behavioral intentions (see Table 4). Social dominance orientation and ethnic intolerance were also statistically significant predictors.

**Table 4:** Hierarchical multiple regression analyses predicting violent behavioral intentions.

					95% CI	
Predictor	Δ*R*^*2*^	b (*SE*)	*β*	*p*	LL	UL
Step 1	.24***					
Social Dominance Orientation		.14 (.06)	.16	.017	.025	.245
Right-Wing Authoritarianism		-.04 (.07)	-.04	.552	-.186	.100
Nationalism		.15 (.08)	.15	.055	-.003	.296
Ethnic Intolerance		.37 (.07)	.15	< .001	.234	.499
Ethnic Identification		-.10 (.06)	-.09	.122	-.220	.026
Step 2	.34***					
Social Dominance Orientation		.15 (.05)	.17	.007	.041	.253
Right-Wing Authoritarianism		.02 (.07)	.02	.771	-.116	.156
Nationalism		.03 (.08)	.03	.734	-.122	.173
Ethnic Intolerance		.26 (.07)	.26	< .001	.126	.389
Ethnic Identification		-.04 (.06)	-.04	.536	-.166	.087
Adventurer		.34 (.06)	.36	< .001	.211	.464
Fellow Traveler		-.04 (.08)	-.03	.599	-.204	.118
Leader		-.06 (.06)	-.06	.335	-.182	.062
Drifter		.13 (.07)	.11	.047	.002	.265
Misfit		-.03 (.08)	-.03	.676	-.196	.127

Note. ***p < .001.

### Criterion/Convergent validity

The different extremist archetypes were expected to be positively correlated with the different ideology, prejudice, and ethnic identification scales. The bivariate correlations supported this assumption (see Table 2). When examining the partial correlations controlling for the remaining archetypes, most results held (see Table 3). In the bivariate correlations, the “fellow traveler” had positive correlations with nationalism and ethnic identification. When controlling for the remaining archetypes, the “fellow traveler” continued to be positively associated with ethnic identification, in addition to new significant negative associations with ethnic intolerance and SDO. Whereas the “leader” had positive bivariate correlations with all of the scales, only the positive associations with SDO and nationalism held when controlling for the remaining archetypes. The “drifter” had positive zero-order correlations with SDO, nationalism, and ethnic intolerance. The positive associations with SDO and nationalism remained significant when examining the partial correlations. The “misfit” continued to have positive correlations with all the ideology scales when controlling for the other archetypes, whereas the associations between the “adventurer” and all variables became statistically nonsignificant when examining the partial correlation.

### Preliminary discussion

A 30-item Extremist Archetypes Scale with five subscales (adventurer, fellow traveler, leader, drifter, and misfit) was developed using EFA. The reliability of the subscales was acceptable to excellent, and the skewness values indicated a normal distribution of all subscales. Even though controlling for established predictors of intergroup hostility (e.g., SDO and RWA), four of the archetype subscales had significant, positive zero-order correlations with violent behavioral intentions, supporting their predictive validity. Furthermore, all five extremist archetype dimensions had different partial correlations with established measures of ideology, prejudice, and ethnic identification. These findings support the convergent validity of the scale but also indicate discriminant validity.

## Study 2

As the results of Study 1 provided initial support for the satisfactory psychometric properties of the Extremist Archetype Scale, in Study 2 we aimed at confirming our findings and further validating the scale using a new sample and new validation measures. For a further test of the scale’s predictive validity, we included a scale on radicalism intentions [43] in addition to the violent behavioral intentions scale from Study 1, as it is one of the most commonly used scales in the field. Moreover, in light of previous research on personality and violence [12] and the personality-like descriptions of the archetypes in Bjørgo [3] and Nesser [4] we aimed to explore the unique personality profiles (i.e., associations) underlying the different archetypes in terms of HEXACO personality inventory (domains and facets) and the Dark Triad personality traits.

### Method

#### Participants

An a priori power analysis showed that a sample size of 303 provided perfect power (1.00) to detect the average effect size observed in Study 1 (*f*^*2*^ = .15). This number also satisfied the rule of thumb of 10 participants per variable in SEM-based analyses [10 participants for each of the 30 retained scale items; 37]. We collected 308 complete responses from White/Caucasian Americans (*M*_age_ = 43.56, *SD*_age_ = 13.24, range = 21–76, 48.5% female). The sample was relatively politically heterogeneous, with a sample mean of 4.64 (*SD* = 2.84) on a 10-point Likert scale ranging from 1 (*very liberal*) to 10 (*very conservative*). A comprehensive overview of the demographics can be found in the SOM.

#### Procedure

An online survey was used to collect data in December 2019. Participants were recruited using the same procedure, payment rate, and ethical considerations as described in Study 1. Respondents from the first study were prevented from participating in this second study, ensuring unique samples. The study was preregistered at https://osf.io/nm8da.

#### Instruments

Unless stated otherwise, responses were rated on 7-point Likert scales, ranging from 1 (*strongly disagree*) to 7 (*strongly agree*). All items can be found in the SOM.

*Extremist archetypes scale*. Participants indicated their agreement with the 30 items that were retained in Study 1. See Appendix A for the scale items and instructions.

*Personality*. To measure the basic personality traits, we used the HEXACO-60 [14], which measures six major dimensions of personality: emotionality (α = .85), extraversion (α = .87), honesty-humility (α = .80), agreeableness (α = .81), conscientiousness (α = .83), and openness (α = .83). Each of the factors has four underlying facets that were computed in addition to the overarching traits. In line with the original instructions, the HEXACO items were measured on a 5-point Likert scale ranging from 1 (*strongly disagree*) to 5 (*strongly agree*).

*Dark Triad*. To measure the Dark Triad of undesirable personality traits, we included the Dirty Dozen Scale [15]. The scale consists of three subscales with four items associated with each subscale (Machiavellianism, α = .84), (Psychopathy, α = .74) and (Narcissism, α = .83).

*Violent intentions*. We use the same scale on violent behavioral intentions [17] that was described in Study 1 (α = .90).

*Radicalism*. The Radicalism Intention Scale assesses readiness to participate in illegal or violent political action [43].

### Analyses

First, to confirm the factor structure of the Extremist Archetypes Scale, confirmatory factor analysis (CFA) using SEM was conducted. To fit the model, we used Mplus 8 [44] employing robust maximum likelihood estimation (MLR) to provide robust standard errors and a scaled test statistic. We then estimated the fit of the five-factor model found in the previous study and compared it to alternative solutions. Because the chi-square test is a less adequate fit estimate for samples with more than 200 cases, the comparative fit index (CFI) and root mean square error of approximation (RMSEA) were used as primary estimates of model fit.

We also tested for correlations between the mean-scored archetypes scales with the violence variables to examine the predictive validity and the correlations with the personality variables to test criterion/convergent validity as in Study 1. Again, we report zero-order and partial correlations

### Results

#### CFA

We compared the five-factor solution from Study 1 to alternative solutions. Most of the research that differentiates between different types of extremists does so by focusing on a leader-follower distinction [e.g., 5]. Hence, we compared the five-factor model to a two-factor model differentiating between the leader (i.e., all leader items loading on the first factor) and the follower (the remaining four archetypes items loading on the second factor). In addition, as the descriptions of the “drifter” and the “fellow traveler” [3,4] could be seen overlapping to a certain degree, a four-factor solution where these two archetypes were treated as one factor was tested. Next, given that extremists often have been viewed as a homogeneous group in previous research, the five-factor model was compared to a one-factorial solution, in which all archetypes loaded on a single factor.

In the five-factor solution, all indicators yielded significant positive factor loadings with standardized coefficients ranging from .43 to .95 (see SOM). The five-factor model fit was relatively satisfactory but not excellent in terms of the fit indices (see Table 5). When reviewing the modification indices, adding a covariance between item 4 (i.e., “Other group members see me as someone who likes thrills and adventure”) and item 5 (i.e., “I like groups that bring along adventure and adrenaline”) from the “adventurer” archetype substantially increased the model fit (MI = 39.54). When adding this covariance, all fit indices improved and consequently met the traditional cutoff values [45,46]. Importantly, both the five-factor model and the same model with the covariance between items 4 and 5 added fit the data better than each of the alternative solutions (see Table 5). Hence, the results supported the five-factor structure found in Study 1. The reliability coefficients were acceptable to satisfactory: “adventurer” (α = .93), “leader” (α = .91), “drifter” (α = .81), “misfit” (α = .78), and “fellow traveler” (α = .71).

**Table 5:** Fit indices for structural equation models.

Model	X^2^	*df*	X^2^/*df*	*p*	CFI	RMSEA	90% CI
5-factor solution	796.5	397	2.0	< .001	.895	.057	[.051, .063]
5-factor solution with covariance between items 4 and 5	757.9	396	1.9	< .001	.905	.054	[.049, .060]
4-factor solution where Drifter and Fellow Traveler are together	1032.7	399	2.6	< .001	.833	.072	[.066, .077]
2-factor solution that differentiates between Leader and Follower	1735.8	404	43	< .001	.650	.103	[.098, .108]
1-factor solution	2217.0	405	5.5	< .001	.524	.121	[.116, .125]

*Note*. CFI = comparative fit index. RMSEA = root mean square error of approximation. Estimator = robust maximum likelihood (MLR). CI = confidence interval for RMSEA.

#### Common method variance

As some of the fit indices were acceptable but not excellent (i.e., CFI), we checked for common method variance (CMV) as a possible explanation. CMV is present when variations in responses are caused by the instrument rather than the actual predispositions of the respondents that the instrument attempts to assess. The results from Harman’s single factor technique [47, calculated variance of 26%] and the common latent factor technique (calculated variance of 46%) indicated little evidence for CMV.

#### Predictive validity

Based on the results from Study 1, we expected the archetypes “adventurer,” “leader,” “drifter,” and “misfit” to be positively correlated with violent intentions. Replicating and extending findings from Study 1, the same four out of five archetypes were related to violent behavioral and radicalization intentions (see Table 6). Notably, there was a positive correlation between the archetype “fellow traveler” (the archetype not showing correlations with violent intentions in Study 1) and radicalism intentions, indicating that this archetype might also be related to a willingness to participate in illegal or violent political action.

**Table 6:** Correlations between the scales.

Scale	1.	2.	3.	4.	5.	6.	7.	8.	9.	10.	11.	12.	13.	14.	15.
1. Adventurer															
2. Fellow Traveler	.17**														
3. Leader	.63***	.20***													
4. Drifter	.22***	.09	.07												
5. Misfit	.35***	.41***	.19***	.31***											
6. Honesty-humility	-.25***	-.19**	-.20***	-.32***	-.35***										
7. Emotionality	-.43***	.22***	-.36***	.01	.02	-.03									
8. Extraversion	.37***	.03	.59***	-.25***	-.09	.01	-.31***								
9. Agreeableness	-.03	-.03	.03	-.28***	-.14*	.31***	-.12*	.34***							
10. Conscientiousness	-.20***	-.01	.10	-.41***	-.41***	.31***	-.10	.28***	.15**						
11. Openness to Experience	.15***	-.05	.22***	-.05	-.28***	.12*	-.13*	.27***	.14*	.31***					
12. Machiavellianism	.28***	.19**	.22***	.44***	.45***	-.67***	.00	-.06	-.32***	-.35***	-.05				
13. Psychopathy	.24***	.05	.10	.45***	.37***	-.43***	-.18**	-.26***	-.53***	-.33******	-.14*	.62***			
14. Narcissism	.38***	.31***	.38***	.24***	.43***	-.52***	-.02	.20***	-.24***	-.16**	-.04	.60***	.40***		
15. Violent Behavioral Intentions	.37***	-.02	.31***	.22***	.30***	-.18**	-.23***	.12*	-.16**	-.22***	-.04	.23***	.29***	.29***	
16. Radicalism Intentions	.34***	.13*	.20***	.26***	.35***	-.21***	-.08	-.01	-.12*	-.29***	-.02	.28***	.28***	.31***	.56***

Note

*p < .05

**p < .01

***p < .001.

To determine the unique relationship between the archetypes and violence measures, partial correlations were estimated for each archetype, controlling for the remaining four archetypes (See Table 7). The “adventurer,” “drifter” and “misfit,” archetypes were all partially correlated with both violence variables. In terms of partial correlations, the “leader” archetype was positively correlated with violent behavioral intentions, but the correlation with radicalism intentions fell below statistical significance. Replicating the findings of Study 1, the “fellow traveler” archetype had negative partial correlations with violent behavioral intentions when controlling for the remaining archetypes. Hence, the findings from Study 2 mostly exhibited the same relationships as those found in Study.

**Table 7:** Psychometric properties and partial correlations of the scales.

					Partial correlations									
					Adventurer		Traveler		Leader		Drifter		Misfit	
Variable	Items	*M*	*SD*	Skew	*r*_p_	*p*	*r*_p_	*p*	*r*_p_	*p*	*r*_p_	*p*	*r*_p_	*p*
Extremist Archetypes														
Adventurer	6	3.39	1.41	.23										
Fellow Traveler	6	4.43	0.90	-.73	-.06	.300								
Leader	6	3.83	1.35	-.06	.61	< .001	.15	.012						
Drifter	6	3.16	1.11	.47	.16	.006	-.04	.539	-.09	.136				
Misfit	6	2.63	0.96	.79	.25	< .001	.38	< .001	-.06	.274	.25	< .001		
Personality														
Honesty-humility	10	3.54	.72	-.17	-.04	.465	-.05	.409	-.08	.168	-.23	< .001	-.20	.001
Emotionality	10	3.15	.78	-.10	-.36	< .001	.30	< .001	-.13	.028	.07	.253	.09	.144
Extraversion	10	3.22	.80	-.20	.16	.005	-.03	.651	.51	< .001	-.33	< .001	-.19	.001
Agreeableness	10	3.31	.68	-.09	.04	.550	.01	.890	.03	.555	-.27	< .001	-.06	.349
Conscientiousness	10	3.85	.63	-.23	-.21	< .001	.17	.003	.29	< .001	-.28	< .001	-.39	< .001
Openness to Experience	10	3.66	.74	-.50	.12	.034	.07	.260	.16	.006	.05	.403	-.39	< .001
Dark Triad														
Machiavellianism	4	2.66	1.31	.71	.02	.789	-.001	.981	.10	.074	.35	< .001	.30	< .001
Psychopathy	4	2.56	1.16	.69	.07	.247	-.11	.050	-.02	.706	.37	< .001	.25	< .001
Narcissism	4	2.81	1.33	.57	.09	.146	.13	.027	.21	< .001	.16	.007	.24	< .001
Violence														
Violent Behavioral Intentions	7	2.58	1.35	.69	.14	.013	-.20	< .001	.15	.010	.14	.019	.20	.001
Radicalism Intentions	4	2.42	1.31	.65	.17	.003	-.01	.817	-.00	.950	.15	.012	.19	.001

*Note*. Potential range for the personality scales are 1–5; the rest range from 1–7. *r*_*p*_ = partial correlations. Control variables in the partial correlations are the remaining archetypes. Traveler = Fellow Traveler.

#### Criterion/Convergent validity

Because we wanted to map the unique associations between the different archetype dimensions and principal domains of personality for further validation of the scale, we conducted partial correlations (controlling for the remaining four archetype dimensions) between each archetype and the HEXACO personality (on both the domain and the facet level) and the Dark Triad dimensions.

The “adventurer” had positive partial correlations with extraversion and openness and negative partial correlations with emotionality and conscientiousness (see Table 7 for all domain-level partial correlations). When looking at the facet level, the “adventurer” further had partial correlations with ten of the facets, with the strongest negative correlations being with fearfulness, prudence, and anxiety (see Table 8 for all facet-level partial correlations). The “adventurer” did not have any partial correlations with the Dark Triad.

**Table 8:** Psychometric properties and partial correlations between the extremist archetypes and the HEXACO facets.

* *	* *	* *	* *	* *	* *	Partial correlations									
						Adventurer		Traveler		Leader		Drifter		Misfit	
Variable	Items	α/*r* ^*a*^	*M*	*SD*	Skew	*r*_p_	*p*	*r*_p_	*p*	*r*_p_	*p*	*r*_p_	*p*	*r*_p_	*p*
Honesty-humility															
Sincerity	3	.73	3.50	0.94	-.19	.11	.057	.01	.882	-.04	.451	-.08	.194	**-.26**	**< .001**
Fairness	3	.85	3.63	1.19	-.59	**-.16**	**.005**	-.04	.538	.05	.378	**-.28**	**< .001**	-.06	.343
Greed-Avoidance	2	.46*	3.09	1.03	-.16	-.02	.739	**-.18**	**.002**	-.06	.312	-.09	.137	.05	.381
Modesty	2	.52*	3.91	0.92	-.72	.00	.985	.10	.099	**-.28**	**< .001**	**-.15**	**.010**	**-.31**	**< .001**
Emotionality															
Fearfulness	3	.73	3.13	0.99	-.20	**-.41**	**< .001**	**.19**	**.001**	-.09	.115	**.13**	**.024**	**.12**	**.043**
Anxiety	2	.64*	3.42	1.14	-.40	**-.24**	**< .001**	**.14**	**.016**	**-.17**	**.003**	**.15**	**.010**	.05	.421
Dependence	2	.48*	2.71	1.01	.17	**-.21**	**< .001**	**.20**	**< .001**	-.09	.121	.06	.313	**.17**	**.004**
Sentimentality	3	.71	3.29	0.93	-.23	**-.19**	**.001**	**.33**	**< .001**	-.03	.567	**-.12**	**.037**	-.06	.336
Extraversion															
Social Self-Esteem	3	.78	3.71	0.93	-.68	.05	.381	-.04	.537	**.31**	**< .001**	**-.27**	**< .001**	**-.25**	**< .001**
Social Boldness	3	.76	3.01	0.97	-.04	**.20**	**.001**	**-.14**	**.018**	**.62**	**< .001**	**-.18**	**.002**	**-.17**	**.003**
Sociability	2	.58*	2.68	1.08	.14	**.15**	**.008**	**.13**	**.027**	**.32**	**< .001**	**-.23**	**< .001**	.07	.238
Liveliness	2	.62*	3.36	1.06	-.39	.09	.112	-.02	.729	**.27**	**< .001**	**-.30**	**< .001**	**-.16**	**.005**
Agreeableness															
Forgiveness	2	.70*	3.10	1.07	-.25	.08	.170	-.02	.697	-.02	.779	**-.13**	**.023**	.04	.540
Gentleness	3	.69	3.25	0.91	-.31	.09	.135	.06	.276	-.06	.286	**-.27**	**< .001**	-.07	.239
Flexibility	3	.42	3.21	0.76	-.01	-.07	.244	-.05	.372	.09	.108	**-.17**	**.004**	.01	.827
Patience	2	.51*	3.74	0.95	-.60	-.01	.870	.03	.610	**.12**	**.043**	**-.22**	**< .001**	**-.16**	**.007**
Conscientiousness															
Organization	2	.41*	3.95	0.84	-.67	**-.17**	**.003**	**.19**	**.001**	**.15**	**.010**	**-.25**	**< .001**	**-.27**	**< .001**
Diligence	2	.39*	4.03	0.80	-.82	.05	.410	.08	.187	**.26**	**< .001**	**-.15**	**.011**	**-.35**	**< .001**
Perfectionism	3	.54	3.66	0.74	-.07	**-.19**	**.001**	**.18**	**.001**	**.21**	**< .001**	-.09	.125	**-.22**	**< .001**
Prudence	3	.73	3.84	0.82	-.62	**-.26**	**< .001**	.06	.277	**.25**	**< .001**	**-.36**	**< .001**	**-.37**	**< .001**
Openness to Experience															
Aesthetic Appreciation	2	.60*	3.67	1.14	-.72	.03	.570	.00	.942	**.12**	**.045**	-.03	.595	**-.23**	**< .001**
Inquisitiveness	2	.33*	3.85	0.94	-.80	.02	.767	.11	.058	**.14**	**.019**	.02	.752	**-.30**	**< .001**
Creativity	3	.75	3.59	0.99	-.55	.07	.214	.07	.211	**.24**	**< .001**	.10	.088	**-.34**	**< .001**
Unconventionality	3	.65	3.58	0.86	-.41	**.21**	**< .001**	.02	.781	-.03	.576	.04	.498	**-.30**	**< .001**

*Note*. *r*_*p*_ = partial correlations. Control variables in the partial correlations are the remaining four archetypes. *p* < .05 are in boldface.

^a^Alpha coefficients are presented for scales with more than two items. Correlation coefficients are presented for two items

*p < .001.

Traveler = Fellow Traveler.

The “fellow traveler” had positive partial correlations with emotionality and conscientiousness. These findings further converged with partial correlations on the facet level. Here, this archetype showed positive associations with sentimentality, dependence, and organization. In addition, the “fellow traveler” dimension showed a positive association with narcissism.

The “leader” dimension had positive partial correlations with extraversion, conscientiousness, and openness. On the facet level, the strongest positive correlations were observed with the facets of the extraversion domain (e.g., social boldness, sociability, and social self-esteem). As with the “fellow traveler,” the “leader” also had a positive association with narcissism. The “drifter” dimension had negative partial correlations with the personality domains of honesty-humility, extraversion, agreeableness, and conscientiousness. These findings are further supported by the partial correlations on the facet level. Here, the archetype had negative associations with liveliness, prudence, and fairness, among others. Moreover, the “drifter” had positive partial correlations with all the Dark Triad dimensions. Finally, the “misfit” dimension had negative partial correlations with honesty-humility, extraversion, consciousness, and openness to experience. On the facet level, the strongest correlations of this archetype dimension were negative associations with prudence, diligence, and creativity (see Table 8). As with the “drifter,” the “misfit” had positive associations with all three Dark Triad personality traits.

### Preliminary discussion

In Study 2, the fit indices supported the five-factor model previously identified in Study 1, which outperformed various alternative solutions. The reliability coefficients were acceptable to satisfactory across all subscales. In support of the scale’s predictive validity, four of the extremist archetypes showed bivariate and positive correlations with the violence variables as in Study 1, and many of these correlations held even when controlling for the four remaining archetypes. The different partial correlations between the archetype dimensions and personality traits supported the notion that the archetype dimensions reflect different personality profiles and therefore unique motivations and behavioral inclinations in joining an extremist group. Although we largely confirmed the psychometric properties of the Extremist Archetype Scale from Study 1, our validation is still limited by our use of a White majority sample. In Study 3, we, therefore, aimed to address this shortcoming by focusing on Muslim minorities and Islamist extremism, broadening our focus beyond right-wing extremism and majority populations.

## Study 3

The aim of Study 3 was to replicate the findings of Study 2 using a sample from an ethnic minority group (i.e., a minority sample of Muslims in the UK). That is, we examined the scale’s factor structure as well as its predictive and criterion validity. We also addressed an additional limitation. The previous two studies assessed the archetypes in terms of roles people they saw as *politically* representing them, whereas violent intentions were assessed in terms of fighting for one’s *ethnic* group. Although the archetypes scale still predicted violent intentions under such circumstances, it is possible that the target group moderates for the strength of effects. As such, in the present study, we tested to what extent the archetypes scale would predict violence on behalf of one’s ethnic group and on behalf of one’s political group.

### Method

#### Participants

The power analysis for Study 2 applies to Study 3, requiring a sample size of 303. Consequently, we sampled 317 Muslims residing in the UK (*M*_age_ = 29.24, *SD*_age_ = 8.59, range = 18–56; 65.4% female, 33.6 male, and 0.9% other). Again, the sample was relatively heterogeneous regarding political orientation, with a sample mean of 4.03 (*SD* = 1.75) on a 10-point Likert scale ranging from 1 (*very liberal*) to 10 (*very conservative*). An overview of the descriptive data can be found in the SOM.

#### Procedure

Data were collected in May 2021 through an online survey by recruiting participants through Prolific using the same procedure, payment rate, and ethical considerations as described in Studies 1 and 2.

#### Instruments

The same measurement scales used in Study 2 were employed with the exception of measuring radicalism and violent intentions one time in terms of one’s ethnic group and one time in terms of one’s political group.

*Extremist archetypes scale*. The scale included the same 30 items used in Studies 1 and 2.

*Personality*. To measure personality traits, we again used the HEXACO-60 [14], consisting of the following subscales: agreeableness (α = .76), conscientiousness (α = .76), emotionality (α = .77), extraversion (α = .80), honesty-humility (α = .76), and openness (α = .75).

*Dark Triad*. The Dirty Dozen Scale [15] was used including three subscales: Machiavellianism (α = .82), Psychopathy (α = .72), and Narcissism (α = .83).

*Violent intentions*. The scale on violent behavioral intentions [17] was assessed in terms of one’s ethnic group (α = .89) and political group (α = .90).

*Radicalism*. The Radicalism Intention Scale [43] was likewise measured one time in terms of one’s ethnic group (α = .86) and one time in terms of one’s political group (α = .85).

### Analysis of data

First, to confirm the factor structure of the Extremist Archetypes Scale in an ethnic minority sample, confirmatory factor analysis (CFA) conducted in Mplus 8 [44] using maximum likelihood estimation with robust standard errors. Similar to Study 2, we estimated the fit of the five-factor model and compared it to the same set of alternative solutions. Next, we investigated correlations of the mean-scored archetypes scales with the violence variables to examine the scale’s predictive validity and with personality variables to examine the scale’s criterion validity.

### Results

#### CFA

As in Study 2, the 5-factor solution showed superior fit (see Table 9). However, as in the previous study, when reviewing the modification indices, adding a covariance between item 4 and item 5 from the “adventurer” archetype substantially increased the model fit (MI = 29.66). Adding this covariance improved fit indices, reaching the traditional cutoff criteria for a satisfactory fit [45,46]. As in Study 2, the reliability coefficients were acceptable to excellent: “adventurer” (α = .90), “leader” (α = .87), “drifter” (α = .73),“misfit” (α = .71), and “fellow traveler” (α = .66).

**Table 9:** Fit indices for structural equation models.

Model	X^2^	*df*	X^2^/*df*	*p*	CFI	RMSEA	90% CI
5-factor solution	729.4	397	1.8	< .001	.892	.051	[.045, .057]
5-factor solution with covariance between items 4 and 5	701.6	396	1.8	< .001	.901	.049	[.043, .055]
4-factor solution where Drifter and Fellow Traveler are together	874.6	400	2.2	< .001	.846	.061	[.056, .067]
2-factor solution that differentiates between Leader and Follower	1436.7	405	3.6	< .001	.665	.090	[.085, .095]
1-factor solution	1620.7	406	4.0	< .001	.605	.097	[.092, .102]

*Note*. CFI = comparative fit index; RMSEA = root mean square error of approximation. Estimator = robust maximum likelihood (MLR). CI = confidence interval for RMSEA.

#### Common method variance

As in Study 2, we examined the possibility for common method variance (CMV). The results from Harman’s single factor technique [47; calculated variance of 24%] and the common latent factor technique (calculated variance of 40%) indicated little evidence for CMV.

#### Predictive validity

Replicating the findings from Studies 1 and 2, we found the “adventurer,” “leader,” “drifter,” and “misfit” dimensions to be positively correlated with violent and radicalism intentions (see Table 10). The were no significant differences between the associations of the ethnic and political violent intentional behavior with the adventurer (*z* = 0.00, *p* = 1.00), fellow traveler (*z* = -1.00, *p* = .317), leader (*z* = -0.99, *p* = .320), drifter (*z* = -0.75, *p* = .456), and misfit dimensions (*z* = -1.78, *p* = .074). Similarly, there were no significant differences between the associations of the ethnic and political radicalism intensions with adventurer (*z* = 1.32, *p* = 0.189), fellow traveler (*z* = -0.33, *p* = .743), leader (*z* = 0.32, *p* = .749), drifter (*z* = -1.91, *p* = .056), and misfit (*z* = -0.98, *p* = .327). In line with Study 2, there was a positive correlation between the archetype “fellow traveler” (the archetype not showing correlations with violent intentions in Study 1) and both violent (ethnic, *r* = .15, *p* < .001; political *r* = .19, *p* < .001) and radicalism intentions (ethnic, *r* = .28, *p* < .001; political *r* = .29, *p* < .001).

**Table 10:** Correlations between the scales.

Scale	1.	2.	3.	4.	5.	6.	7.	8.	9.	10.	11.	12.	13.	14.	15.	16.	17.
1. Adventurer																	
2. Fellow Traveler	.38**																
3. Leader	.71***	.33***															
4. Drifter	.22***	.31***	.26***														
5. Misfit	.29***	.51***	.27***	.35***													
6. Honesty-humility	-.22***	-.29***	-.30***	-.36***	-.31***												
7. Emotionality	-.24***	.05	-.11	-.05	-.01	-.01											
8. Extraversion	.39***	-.05	.48***	-.13*	-.07	-.02	-.15**										
9. Agreeableness	-.02	-.03	.06	-.29***	-.06	.32***	-.04	.18**									
10. Conscientiousness	-.10	-.20***	.00	-.31***	-.33***	.37***	.03	.20***	.20**								
11. Openness	.17**	-.05	.21***	.06	-.23***	-.01	.00	.24***	.15*	.24***							
12. Machiavellianism	.22***	.23***	.25***	.32***	.36***	-.60***	-.05	-.03	-.26***	-.43***	-.09						
13. Psychopathy	.15**	.26***	.16**	.38***	.38***	-.47***	-.22***	-.19**	-.38***	-.39***	-.24***	.62***					
14. Narcissism	.21***	.28***	.30***	.29***	.30***	-.54***	.07	.08	-.21***	-.25***	.01	.54***	.47***				
15. Violent Behavioral Intentions ethnic	.22***	.15**	.12*	.13*	.21***	-.29***	-.26***	-.04	-.11*	-.24***	-.09	.31***	.34***	.15**			
16. Violent Behavioral Intentions political	.22***	.19**	.16**	.16**	.28***	-.32***	-.24***	-.10	-.08	-.33***	-.16**	.34***	.40***	.19**	.74***		
17. Radicalism Intentions ethnic	.31******	.28***	.20***	.13*	.26***	-.30***	-.15**	-.02	-.15**	-.26***	-.04	.24***	.24***	.11	.60***	.58***	
18. Radicalism Intentions political	.27***	.29***	.19**	.19**	.29***	-.33***	-.14*	-.06	-.13*	-.29***	-.06	.27***	.31***	.12*	.62***	.61***	.84***

Note

*p < .05

**p < .01

***p < .001.

Similar to Study 2, the unique relationships between the archetypes and the measures of extremism were determined through partial correlations, controlling for the remaining four archetypes (See Table 11). The “adventurer,” “traveler,” and “misfit” archetypes were all partially correlated with higher scores on one or more of the violence variables. In terms of partial correlations, both the “adventurer” and “misfit” archetypes were positively correlated with both violent behavioral and radicalism intentions (both ethnic and political). Furthermore, the “fellow traveler” archetype yielded partial correlations with radicalism but not with violent behavioral intentions when controlling for the remaining archetypes.

**Table 11:** Psychometric properties and partial correlations of the scales.

					Partial correlations									
					Adventurer		Traveler		Leader		Drifter		Misfit	
Variable	Items	*M*	*SD*	Skew	*r*_p_	*p*	*r*_p_	*p*	*r*_p_	*p*	*r*_p_	*p*	*r*_p_	*p*
Extremist Archetypes														
Adventurer	6	3.93	1.23	-.24										
Fellow Traveler	6	4.21	0.88	-.39	.18	< .001								
Leader	6	3.82	1.14	.13	.66	< .001	.03	.579						
Drifter	6	3.31	0.98	.08	-.02	.734	.13	.027	.13	.023				
Misfit	6	2.66	0.91	.35	.05	.429	.41	< .001	.04	.497	.22	< .001		
Personality														
Honesty-humility	10	3.56	.65	-.36	.05	.371	-.09	.121	-.17	.003	-.24	< .001	-.12	.028
Emotionality	10	3.35	.61	-.35	-.26	< .001	.15	.009	.09	.122	-.05	.374	-.01	.904
Extraversion	10	3.17	.63	-.24	.16	.005	-.16	.005	.38	< .001	-.23	< .001	-.11	.059
Agreeableness	10	3.29	.59	-.12	.04	.514	.04	.461	-.03	.602	-.29	< .001	-.02	.730
Conscientiousness	10	3.57	.56	-.16	-.09	.122	-.02	.726	.17	.003	-.24	< .001	-.23	< .001
Openness to Experience	10	3.39	.64	-.12	.08	.179	-.01	.884	.16	.005	.11	.061	-.30	< .001
Dark Triad														
Machiavellianism	4	2.32	1.18	.93	.02	.768	-.00	.949	.08	.121	.12	.001	.23	< .001
Psychopathy	4	2.41	1.05	.82	.00	.980	.03	.555	.0	.997	.28	< .001	.24	< .001
Narcissism	4	3.02	1.37	.32	-.05	.366	.10	.083	.18	.002	.15	.007	.13	.018
Violence														
Violent Behavioral Intentions ethnic	7	2.28	1.27	.79	.12	.035	-.00	.973	-.07	.209	.05	.384	.13	.027
Violent Behavioral Intentions political	7	2.12	1.17	.80	.17	.003	.02	.750	-.03	.614	.05	.368	.18	.001
Radicalism Intentions ethnic	4	2.71	1.51	.60	.20	< .001	.11	.043	-.05	.371	.01	.877	.12	.042
Radicalism Intentions political	4	2.53	1.40	.64	.14	.011	.12	.032	-.04	.532	.06	.257	.13	.018

*Note*. Potential range for the personality scales are 1–5; the rest range from 1–7. *r*_*p*_ = partial correlations. Control variables in the partial correlations are the remaining archetypes. Traveler = Fellow Traveler.

#### Criterion validity

Replicating Study 2, we examined the partial correlations (controlling for the remaining four archetype dimensions) between each archetype and the HEXACO personality (on both the domain and the facet level) and the Dark Triad dimensions.

The “adventurer” had positive partial correlations with extraversion and negative partial correlations with emotionality (see Table 11 for all domain-level partial correlations). When looking at the facet level, the “adventurer” further had partial correlations with eight of the facets, with the strongest negative correlations being with fearfulness, anxiety, and prudence (see Table 12 for all facet-level partial correlations). Similar to the results of Study 2, the “adventurer” did not have any partial correlations with the Dark Triad.

**Table 12:** Psychometric properties and partial correlations between the extremist archetypes and the HEXACO facets.

* *	* *	* *	* *	* *	* *	Partial correlations									
						Adventurer	Traveler	Leader	Drifter	Misfit		
Variable	Items	α/*r* ^*a*^	*M*	*SD*	Skew	*r*_p_	*p*	*r*_p_	*p*	*r*_p_	*p*	*r*_p_	*p*	*r*_p_	*p*
Honesty-humility															
Sincerity	3	.60	3.55	0.86	-.29	.08	.153	-.09	.107	-.06	.322	**-.16**	**.005**	-.07	.214
Fairness	3	.73	3.37	0.39	-1.15	.01	.875	-.09	.120	-.07	.226	**-.14**	**.016**	**-.16**	**.006**
Greed-Avoidance	2	.43*	2.96	0.98	-.01	.06	.305	-.10	.092	**-.15**	**.007**	**-.17**	**.003**	.06	.272
Modesty	2	.41*	3.69	0.87	-.33	-.03	.656	.09	.102	**-.24**	**< .001**	**-.12**	**>.010**	**-.15**	**.010**
Emotionality															
Fearfulness	3	.55	3.25	0.79	-.14	**-.29**	**< .001**	**.14**	**.015**	.06	.332	-.08	.172	.04	.537
Anxiety	2	.44*	3.67	0.85	-.30	**-.26**	**< .001**	**.19**	**.001**	.08	.150	.05	.390	-.0	.252
Dependence	2	.36*	2.88	0.92	-.21	**-.17**	**.003**	.06	.255	.09	.102	.06	.278	.03	.614
Sentimentality	3	.62	3.55	0.78	-.47	-.07	.209	.06	.298	.04	.499	**-.13**	**.018**	-.03	.623
Extraversion															
Social Self-Esteem	3	.66	3.32	0.85	-.32	.06	.311	-.09	.126	**.29**	**< .001**	**-.25**	**< .001**	**-.15**	**.009**
Social Boldness	3	.66	2.96	0.80	-.12	**.13**	**.027**	**-.16**	**.005**	**.38**	**< .001**	-.07	.254	-.07	.247
Sociability	2	.36*	3.14	0.92	-.27	**.17**	**.002**	**-.15**	**.010**	**.18**	**.001**	-.06	.273	.06	.317
Liveliness	2	.36*	3.26	0.78	-.25	.11	.058	-.05	.372	**.22**	**< .001**	**-.26**	**< .001**	**-.12**	**.032**
Agreeableness															
Forgiveness	2	.67*	3.17	1.04	-.20	.07	.238	-.02	.728	-.01	.826	**-.22**	**< .001**	**.12**	**.031**
Gentleness	3	.45	3.38	0.68	-.22	**.13**	**.024**	.04	.484	-.08	.161	**-.19**	**.001**	-.03	.598
Flexibility	3	.34	3.18	0.68	.03	-.05	.405	.03	.633	.01	.852	**-.18**	**.002**	.01	.891
Patience	2	.57*	3.43	0.95	-.29	-.05	.399	.08	.167	.00	.957	**-.26**	**< .001**	-.05	.358
Conscientiousness															
Organization	2	.26*	3.70	0.81	-.29	-.07	.216	-.03	.561	**.13**	**.028**	**-.19**	**.001**	**-.14**	**.012**
Diligence	2	.33*	3.77	0.77	-.64	.10	.080	-.05	.416	**.14**	**.016**	-.11	.063	**-.25**	**< .001**
Perfectionism	3	.48	3.56	0.70	-.27	-.03	.652	.04	.442	**.15**	**.007**	**-.17**	**.003**	**-.12**	**.031**
Prudence	3	.68	3.38	0.79	-.19	**-.20**	**< .001**	-.03	.541	.07	.211	**-.21**	**< .001**	**-.17**	**.003**
Openness to Experience															
Aesthetic Appreciation	2	.45*	3.14	1.12	-.19	-.02	.759	-.03	.658	**.15**	**.010**	.11	.062	**-.20**	**< .001**
Inquisitiveness	2	.25*	3.66	0.85	-.34	-.02	.714	-.01	.907	**.17**	**.004**	.02	.699	**-.27**	**< .001**
Creativity	3	.67	3.43	0.91	-.22	.09	.086	-.06	.326	**.13**	**.024**	**.15**	**.010**	**-.19**	**.001**
Unconventionality	3	.35	3.33	0.70	.21	**.13**	**.018**	.08	.157	.01	.815	-.01	.934	**-.22**	**< .001**

*Note*. *r*_*p*_ = partial correlations. Control variables in the partial correlations are the remaining four archetypes. *p* < .05 are in boldface.

^a^Alpha coefficients are presented for scales with more than two items. Correlation coefficients are presented for two items

*p < .001.

Traveler = Fellow Traveler.

The “fellow traveler” had positive partial correlations with emotionality and negative partial correlations with extraversion. On the facet level, “fellow traveler” yielded positive associations with anxiety and was negatively linked with social boldness and sociability.

The “leader” dimension had positive partial correlations with extraversion, conscientiousness, and openness as well as negative correlations with honesty-humility. Similar to Study 2, the strongest positive correlations of the “leader” dimension on the facet level were within the extraversion domain (social boldness, liveliness, and social self-esteem). Additionally, the “leader” also had a positive association with narcissism.

Replicating Study 2, the “drifter” dimension had negative partial correlations with the personality domains of honesty-humility, extraversion, agreeableness, and conscientiousness. On the facet level, the archetype had negative associations with liveliness, social self-esteem, and prudence. Moreover, the “drifter” had positive partial positive correlations with all the Dark Triad dimensions.

Finally, the “misfit” dimension had negative partial correlations with honesty-humility, extraversion, and openness. On the facet level, the strongest correlations of this archetype dimension were negative associations with inquisitiveness, prudence, and unconventionality (see Table 11). Like Study 2, the “misfit” had positive associations with all three Dark Triad personality traits.

### Preliminary discussion

In Study 3, we largely replicated the findings of Study 2 in a Muslim minority sample. The fit indices supported the five-factor model previously identified, and the archetype dimensions’ reliability coefficients were all acceptable. There were no significantly different associations of the political and ethnic distinctions of violent behavioral intentions and radicalism intentions with the five extremist archetypes. This finding indicates that the extremist archetypes predict violent intentions regardless of whether these are framed in terms of one’s ethnic or political group. In support of the scale’s predictive validity, three of the extremist archetypes showed bivariate and positive correlations with the violence variables.

## Measurement invariance test

In Studies 1, 2, and 3 we examined and confirmed the facture structure of the Extremist Archetype Scale using both majority and minority samples. In the following section, we examined the scale’s measurement equivalence across gender, age, and political orientation as well as across the samples’ ethnicity/group status (i.e., majority and minority samples). That is, we tested whether the Extremist Archetype scale measures the same psychological construct and shares the same meaning across these demographic and sample differences.

### Method

#### Procedure

All participants from Studies 1 (*N* = 307), 2, (*N* = 308) and 3 (*N* = 317) were pooled into one dataset (*N* = 932; *M*_*age*_ = 37.42, *SD*_*age*_ = 15.04, range = 18–76; 56.0% female, 43.3 male, and 0.6% other).

#### Instruments

We included the Extremist Archetype scale for invariance testing.

### Analysis of data

To test whether the Extremist Archetype scale had the same meaning for different groups, we examined, across the three samples, measurement equivalence of gender, age, and political orientation. In doing so, we used Mplus 8 [44] employing maximum likelihood estimation with robust standard errors. We used median splits to group participants by age (*Mdn* = 36) and political orientation (*Mdn* = 4). The assumption of measurement equivalence was tested through configural, metric, and scalar invariance [48]. Configural invariance assumes that the same scale structure fits both groups (e.g., male and female) with all parameters freely estimated in each group. Metric invariance requires that factor loadings can be constrained to be equal across groups without a significant decrease in model fit. If metric invariance is achieved, comparative analysis of covariance can be validly conducted. Scalar invariance requires intercepts to be constrained to equality across groups without significant decrease in model fit. Scalar invariance allows for mean comparison across groups, indicating that significant differences are not due to differences in scale properties [49,50]. The null hypothesis of metric and scalar invariance was evaluated by comparing the fit indices between the constrained and unconstrained models using Little’s [51] criteria (ΔCFI ≤ .010 and ΔRMSEA ≤ .010).

### Results

First, we examined the measurement equivalence for the five-factor solution with regard to gender. Model fit was acceptable for the configural invariance, χ^2^/df = 2.26; CFI = .904; RMSEA = .052, 90% CI = [.049; .055]. Comparing an unconstrained model against a constrained model with factor loadings set equally across groups (metric invariance) did not yield noteworthy differences in fit indices regarding gender; ΔCFI = .002; ΔRMSEA < .001. Furthermore, comparing the metric invariance model against a constrained model with both factor loadings and intercepts constrained to equality (scalar invariance) likewise did not yield noteworthy differences in fit indices regarding gender ΔCFI = .005; ΔRMSEA < .001.

Second, we examined the measurement equivalence with regard to age. Model fit was acceptable for the configural invariance, χ^2^/df = 2.31; CFI = .900; RMSEA = .053, 90% CI = [.050; .056], and this fit did not deteriorate for the metric (ΔCFI < .001; ΔRMSEA = .001) and scalar (ΔCFI = .009; ΔRMSEA = .002) invariance models.

Third, we examined the measurement equivalence with regard to political orientation. Model fit was acceptable for the configural invariance, χ^2^/df = 2.21; CFI = .909; RMSEA = .051, 90% CI = [.048; .054], and this fit did not deteriorate for the metric (ΔCFI < .001; ΔRMSEA = .001) and scalar (ΔCFI = .002; ΔRMSEA < .001) invariance models.

Finally, we examined the measurement equivalence across the three samples (i.e., ethnic minority in Study 3 and ethnic majority in Studies 1 and 2). Model fit was acceptable for the configural invariance, χ^2^/df = 1.90; CFI = .899; RMSEA = .054, 90% CI = [.051; .057]. Furthermore, model fit did not deteriorate for the metric invariance model (ΔCFI = .003; ΔRMSEA < .001). However, the results for the scalar invariance were inconclusive: ΔCFI = .021; ΔRMSEA = .004. Consequently, we examined each item for invariance across samples by constraining one intercept at a time and examining the change in the CFI and RMSEA. None of the paths was found to violate the assumption of invariance (ΔCFI & ΔRMSEA ≤ .010).

### Preliminary discussion

By conducting invariance tests, we established overall support, across the three study samples (i.e., ethnic majority and minority status), for measurement equivalence regarding gender, age, and political orientation. Whereas it would have been optimal to keep constant the country of investigation across the three studies, we opted for recruiting Muslims from the U.K. in the last study, given the low percentage of Muslims among the Mturk population. Nevertheless, measurement invariance tests suggested few differences across the countries. The results indicate that the Extremist Archetypes Scale measures the same construct across age, gender, and political groups. Also across ethnic groups, the scale achieved a high level of measurement invariance, but the scalar invariance was somewhat inconclusive. Hence, the results demonstrate that the scale taps into the same factors structure among White Americans and Muslim minority-group members, but results between these groups should be compared with some caution.

## Discussion

The present research aimed to develop the Extremist Archetype Scale and to validate it in terms of individual-level factors of inclinations toward violent extremist intentions (Studies 1 through 3), sociopolitical ideological orientation and group membership (Study 1), and personality, including HEXACO and the Dark Triad dimensions (Studies 2 and 3). The five-factor solution identified in Study 1 outperformed the various alternative solutions tested in Studies 2 and 3 among samples of majority and minority members, quantitatively supporting our integrative account of the qualitative frameworks of Bjørgo [3] and Nesser [4]. Measurement equivalence was further established regarding gender, age, and political orientation as well as across the three samples consisting of either majority or minority populations from different contexts.

When examining the bivariate associations between the archetype dimensions, most of them were positively correlated. Hence, this indicates that the archetypes are not necessarily mutually exclusive and should instead be viewed as dimensions. As such, a person may theoretically score high on more than one dimension.

Although the archetype dimensions were statistically distinct and showed unique associations with validation measures, they also seemed to share certain attributes. These common attributes for violent extremism, in general, are in line with previous research that finds many common attributes (e.g., identity, seeking belonging) in both violent and nonviolent extremists [52].

### The extremist archetypes and their individual differences

In line with the theoretical and qualitative work of Bjørgo [3] and Nesser [4], five distinct extremist archetype dimensions were operationalized and statistically identified in all three studies. Due to the large number of tests and comprehensive data, we will discuss the associations in terms of the more robust partial correlations, focusing on the unique findings for each archetype.

#### The “Adventurer”

The adventurer had robust positive associations with both violent behavioral intentions and radicalism intentions, supporting the potentially violent aspect of this archetype dimension. As expected we found positive correlations with extraversion and openness to experience, which further confirms that the adventurer joins a group for the adrenaline and the thrill of executing extremist actions themselves. Indeed, none of the ideology, prejudice, ethnic identification, or Dark Triad variables was associated with this archetype dimension. These findings further support the notion that the “adventurer” is primarily involved in violent extremism because of the possibility for action. The “adventurer” further had negative partial correlations with emotionality and conscientiousness, suggesting that people scoring high on these dimensions are not necessarily deterred by the prospect of psychological harm and may feel little worry in stressful situations [53]. Such an interpretation is further supported by findings at the facet level. The four strongest partial correlations indicated that the “adventurer” is tough (fearfulness), acts on impulse (prudence), experiences little stress in response to difficulties (anxiety), and is receptive to ideas that might seem radical (unconventionality). Taken together, the findings are in line with Bjørgo’s [3] description of the adventurer as an individual fascinated by violence, fighting, and the possibility of being a heroic fighter.

#### The “fellow traveler”

Interestingly, the “fellow traveler” dimension negatively correlated with violent behavioral intentions in terms of partial correlations among the majority population. This is somewhat contrary to Bjørgo’s [3] understanding of the “fellow traveler” as someone easily led into militant activities due to their need for acceptance and recognition. Indeed, the “fellow traveler” had a moderate positive partial correlation with ethnic identification, indicating that group membership is of importance to people scoring high on this archetype dimension. However, in Study 3 we found positive correlations between the “fellow traveler” dimension and radicalism intentions. These contradictive findings could indicate that the deprivation and stigmatization that Muslims experience in the West may strengthen their identification with their group [54], which in turn may intensify their behavioral reaction to this perceived injustice and increase the likelihood of engaging in collective action to defend their group [7,55,56]. Such a process may not be in play among high-power majority members.

Partial correlations between the “fellow traveler” dimension and ideological variables suggest that people scoring high on the archetype are indeed more egalitarian and less intolerant, differentiating them from many of the other archetypes. When investigating the relationship with personality traits, this interpretation is supported. In terms of personality associations, the “fellow traveler” had positive partial correlations with emotionality and conscientiousness, indicating that people scoring high on these dimensions may rely on emotional support from others [53]. Further, this is in line with transnational studies on extremism showing that emotionality is negatively associated with violent intentions [12].

This interpretation was further supported by the personality facets, suggesting that people scoring high on the “fellow traveler” dimension may feel strong emotional bonds with others (sentimentality), want to share their difficulties with those who will provide comfort (dependence), tend to seek order (organization), and are inclined to avoid physical harm (fearlessness). In addition, the “fellow traveler” dimension had a positive association (Study 2) and marginally positive association (Study 3) with narcissism; this supports the descriptions of Bjørgo [3] that the “fellow traveler” joins an extremist group for intrapersonal reasons and becomes radicalized as a consequence of joining such a group rather than their radicalization being the reason for joining.

#### The “leader”

When considering the associations between violent intentions and the “leader” archetype dimension, the findings of the three studies only partly converged. In contrast to Study 1, a partial relationship with violent intentions was found in Study 2. However, a partial correlation between the “leader” and radicalism intentions was not found in Studies 2 and 3. These observations are somewhat contradictory, and future research is needed to clarify them. However, they may highlight the different roles of being a leader in a violent extremist group. Leaders may be less consistently violent themselves but are in a position of power where they can command other members to execute violent actions on their behalf. These results are in line with research that finds followers to be more motivated to use violence than leaders [5], especially when the leader instructs them to do so [57].

When examining relationships with the ideology variables, the “leader” dimension was found to have positive partial correlations with SDO and nationalism, indicating that people scoring high on this dimension prefer group-based hierarchy [38] and hold nationalist beliefs [36]. As one may have expected based on previous research, the “leader” had positive partial correlations with extraversion, conscientiousness, and openness to experience. These personality traits indicate that the “leader” shows confidence when leading or addressing groups of people, works in a disciplined way toward goals while carefully deliberating decisions, and takes an interest in unusual ideas or people. These findings overlap closely with general research on leadership qualities [for a review, see 58].

Our interpretation is further supported when examining the partial correlations of the “leader” on the facet level and those of the Dark Triad. The “leader” scored high on narcissism and on all the extraversion and conscientiousness facets, with the highest scores on social boldness (e.g., high confidence within a variety of social situations) and sociability (e.g., enjoy talking to and visiting others). These personality traits might be expected to make it easy for a leader to approach possible recruits and persuade them to join the extremist group. In addition, the “leader” dimension showed a negative relationship with the modesty facet of the honesty-humility trait, indicating that they see themselves as superior and entitled to privileges. Taken together, these findings support the descriptions given by Bjørgo [3] and Nesser [4], who characterize the “leader” as a smart, charismatic individual who is driven by political and ideological motives and may radicalize others.

#### The “drifter”

The “drifter” dimension was the archetype dimension with some of the strongest associations with the two violence variables, indicating that drifters are particularly willing to support a violent extremist group and use violence themselves. This finding is somewhat contradictory to the description by Nesser [4], who states that the “drifter” plays a rather peripheral role in attacks. As with the “leader,” the “drifter” also had positive partial correlations with SDO and nationalism. Hence, our findings further contradict Nesser’s [4] understanding of the “drifter” as an individual who is not religiously or politically oriented.

When examining relationships between the “drifter” archetype and the personality dimensions, several patterns are worth discussing. First, as one may have expected, the “drifter” had negative partial correlations with honesty-humility, agreeableness, and conscientiousness. Nevertheless, we also found negative partial correlations with extraversion. These findings indicate that individuals belonging to this archetype may prefer to stay in the background, retain negative feelings toward others, and make decisions on impulse [53]. Second, when examining personality traits at the facet level, this interpretation is further supported. The “drifter” dimension had negative associations with all four facets of extraversion and agreeableness, with the strongest negative correlations to liveliness (e.g., tends not to feel cheerful or dynamic), gentleness (e.g., critical in their evaluations of others), and social self-esteem (e.g., sense of personal worthlessness). Other facets worth mentioning regarding the “drifter” are the negative associations with prudence (e.g., acts on impulse and tends not to consider consequences) and fairness (e.g., willing to gain by cheating or stealing). Parallel to this, the “drifter” had positive associations with all three of the Dark Triad dimensions, indicating that individuals scoring high on this archetype dimension are willing to manipulate others to get their way, experience low levels of empathy, and admire themselves [15]. As such, it makes sense that people scoring high on this archetype dimension have been argued to rapidly change their group membership depending on whether it helps them fulfill their egocentric needs. Indeed, these self-centered characteristics may explain why the “drifter” rarely holds a trusted position in the cell to which they belong [4].

#### The “misfit”

Analogous to the “leader,” partial correlations of the “misfit” archetype dimension with violence seemed less robust. That is, the “misfit” archetype dimension was related to more violent intentions in terms of zero-order correlations in Study 1 yet in terms of partial correlations only in Studies 2 and 3. Thus, future research is needed to replicate these. Nevertheless, despite these inconsistencies, the positive partial correlations from Studies 2 and 3 together with the zero-order correlations from Study 1 coincide with the descriptions given by Bjørgo [3] and Nesser [4].

The “misfit” was the only archetype dimension that showed positive associations with all the ideology, prejudice, and ethnic identification variables. The strongest associations were with ethnic intolerance and RWA, indicating that the group belongingness aspect and authoritarianism are of significant importance for people scoring high on the “misfit” dimension. These findings contrast with Bjørgo’s [3] description of the misfit as an individual without ideological orientation. However, as described by Bjørgo [3] and Nesser [4], the “misfit” may previously have struggled to “fit in” and has a perception of being treated unjustly. Joining an extremist group can provide a feeling of belongingness. At the same time, a common enemy outlined by the group might give the “misfits” a purpose in life by retaliating against the people who previously treated them badly. Thus, the “misfit” seems to be more invested in the group than, for example, the “drifter” and is more committed to its cause.

In line with what one may have expected, the “misfit” was negatively associated with honesty-humility, extraversion, and conscientiousness. It is important to note that the “misfit” shared many similarities with the “drifter” when it came to the major personality domains (e.g., low scores on honesty-humility, extraversion, and conscientiousness). However, when considering openness to experience, both archetypes differed, as the “misfit” had strong negative partial correlations, whereas the “drifter” had no significant partial correlations. These associations imply that the “misfit” feels little attraction toward ideas that may seem radical or unconventional [53]. The strongest associations on the facet level further support this picture, suggesting that the “misfit” may act on impulse (low prudence), have little self-discipline (low diligence), show little inclination for original thought (low creativity), and see themselves as superior (low modesty). Furthermore, the “misfit” had positive associations with the three Dark Triad dimensions, and this fits with the low score on honesty-humility [59].

In sum, these findings are in line with the descriptions by Bjørgo [3] and Nesser [4], who both described the “misfit” as a seeker of action and excitement who finds roles in an extremist group in which they receive recognition for their violent and criminal competence. Because violence attracts attention, violent actions likely make “misfits,” who otherwise feel ignored or insignificant, feel noticed and powerful [5].

### Strengths, limitations, and future research

In the current investigation, we focused on group-based extremism. However, future studies may also profitably investigate the different archetypes in the context of lone extremists. Based on comparative case studies, Knight et al. [60] documented certain psychological vulnerabilities among lone extremists that overlap with vulnerabilities found among different archetypes. For instance, lone extremists often have previous experiences of marginalization, prejudice, and exclusion leading to feelings of injustice with corresponding action tendencies. Similarly, the misfit archetype has been described as someone who harbors grievances and as someone who feels treated unjustly. Further, Knight et al. [60] showed that lone extremists compared to group-based extremists struggle with feelings of low self-esteem, inferiority, exclusion, and a lack of belonging due to problems regarding social interactions with others. This aligns with the description of the drifter archetype that describes a person who generally struggles with social self-esteem, personal worth, and maintaining positive social interaction with others.

Future research should also test whether our archetypes measure specifically predicts violent extremist intentions or all types of pro-group behaviors. This is important because psychological research has shown how commitments to normative and non-normative action on behalf of one’s group have different social psychological and personality antecedents [7,61]. For instance, whereas people who are more dogmatic, less empathic, and less prone to experience fear and anxiety are more supportive of violence in defense of their groups, people who score higher on empathy and altruism tend to endorse nonviolent means to defend their group [12].

Our data were cross-sectional and cannot speak to causality. Future research is thus needed to test the stability vs. change of scores on the archetype dimensions over time as well as experimentally how they moderate reactions to different situational cues (e.g., those that typically can increase violent intentions). In addition, we relied on self-report measures in the three studies, and this raises concerns about demand characteristics and social desirability [62]. This is a general limitation of research on extremism [63], as it is often not ethically defensible or possible to assess actual violent behavior. However, we find it remarkable that the archetype dimensions emerged in samples from the general population (i.e., across majority and minority), were normally distributed, and were relatively consistently related to more violent behavioral and radicalism intentions.

Although our dependent variable (violent intentions) was validated in a sample of former extremists, a related limitation of the present project is that the typologies of Bjørgo [3] and Nesser [4] were developed based on data from actual extremists, whereas we tested them in the general population. As such, our results need to be validated among previous or active extremists to establish their ecological validity. Future research should also investigate the differences between violent and nonviolent extremist outcomes to establish whether the archetypes predict one, the other, or both types of extremism.

Given the strength of using continuous scores in quantitative research, we decided to assess the archetypes on continuous dimensions rather than categorically assigning participants to one single archetype because people in theory may be characterized by several archetypes, albeit to different degrees. Indeed, recent research on personality traits and violent extremism has shown that extremist violent intentions can be associated independently with multiple nonclinical personality dimensions [12]. Nevertheless, our approach is not without limitations. Conceptually, one may argue that the original archetype framework on which our research was based assigns one archetype per person. Future research using analytic techniques such as cluster or latent profile analyses may help identify whether specific archetypes or rather combinations of them best describe the majority of individuals.

The use of participant samples drawn from online panels could potentially affect the results. Some researchers assume that data from online panels are of lower quality and less representative than data obtained through conventional means [e.g., 64–66]. However, research examining the validity of such data indicates that these differences are small and that online panels often produce more representative data than, for instance, data from student samples [67–69]. Online panels also allow for the selection of specific target demographics, such as ethnicity and religion as employed in the present research. Nevertheless, future studies should test our hypothesis in representative samples to reveal whether the findings obtained in the present studies can be generalized to other populations.

Finally, the association between the archetypes and violent intentions and radicalism could be described as modest. A possible explanation could be that we tested our hypotheses among the general populations who may be reporting a lower level of intentions than actual extremists [70]. However, these associations are no lower than that of other psychological variables (e.g., group identification) and violent intentions [7,55].

Ultimately, the study of archetypes could be valuable for counter-extremism and risk assessment efforts. For example, specific information about the psychological traits and profiles that characterize the different archetypes could help guide the development of individually tailored interventions to counteract violent extremism. For instance, according to Bjørgo [3], the “fellow travelers” are easily led into militant activities due to their need for belonging, acceptance, and recognition. Previous research shows that people report reduced prejudice toward outgroup members when they feel included, given the chance to voice their opinions, which increases their sense of worth and belonging [71–73]. Counter-extremism policies could focus on developing social initiatives to foster and promote social inclusion, thereby minimizing risks of alienation and vulnerability to extremism as indexed by our archetypes measure.

### Conclusions

Previous work has often disregarded the heterogeneity that exists among extremists [1,2]. In this paper, we developed the Extremist Archetypes Scale: based on qualitative work, this scale quantitatively measured different archetype dimensions that may reflect different motivations for joining extremist groups and obtaining different roles within them. Supporting their distinctiveness, these archetype dimensions emerged across different samples and had different personality profiles and associations with violent extremist intentions, ideology, prejudice, and related factors. The study, therefore, supports the notion that there is substantial within-group heterogeneity in extremism that can be quantitatively assessed and provides a measure to explore this heterogeneity in future research.

## Supporting information

S1 File (DOCX)
