# Correction: Measuring Extremist Archetypes: Scale Development and Validation

**DOI:** 10.1371/journal.pone.0296237

**Published:** 2023-12-18

**Authors:** The PLOS ONE Staff

There are errors in the formatting of Tables 1, 4, 7, 10 and 12; and the values are not aligned properly. In Table 6, the correlation coefficient value in column 10, row 13 is incorrect. The correct value is -.33***. The publisher apologizes for these errors. Please see the correct Tables 1, 4, 6, 7, 10 and 12 below.

**Table 1:** Factor Loadings for Exploratory Factor Analysis with Direct Oblimin Rotation.

Item	Adventurer	Traveler	Drifter	Leader	Misfit
46. I am known as a group member that is not afraid to take any risks.	**.88**	-.05	-.03	.05	.08
45. I am known as a group member that is not afraid of facing dangers.	**.85**	-.07	-.01	.05	.09
44. I am willing to take more risks than other people in my group.	**.81**	-.13	-.09	.03	-.07
48. Other group members see me as someone who likes thrills and adventure.	**.80**	-.05	.02	.03	.02
43. I like groups that bring along adventure and adrenaline.	**.79**	.11	-.08	-.06	.06
49. I like to show other group members that I am not afraid of taking risks.	**.73**	-.02	-.03	.12	-.14
15. I try to find a group that accepts me.	-.12	**.64**	-.16	.08	.02
19. To belong to a group, I am willing to do what is asked of me.	.24	**.55**	.19	.04	-.11
13. I tend to "go with the flow" in group settings.	-.01	**.52**	-.07	-.29	.00
40. The group I belong to gives me stability in life.	.03	**.50**	.19	.26	-.24
25. I often join groups that others have introduced me to.	.02	**.42**	-.09	.21	-.18
24. I stay in a group as long as they give me what I need.	-.11	**.41**	-.20	.17	-.12
27. Sometimes, I suddenly decide to leave a group and seek a new one.	.11	.03	**-.66**	-.02	.05
35. Throughout my life I have found it difficult to find a group to belong to.	-.01	.02	**-.61**	-.16	.03
28. It has happened that I just replaced one group I belonged to with another.	.03	.04	**-.59**	.13	-.10
23. I rarely stay with one group for a long period of time.	.04	-.18	**-.59**	-.02	-.08
22. I have shifted a lot between groups in my life.	.05	.13	**-.59**	.16	-.04
47. I get bored more easily than other group members.	.02	-.16	**-.48**	.04	-.15
11. Most group members see me as a leader.	.15	-.01	-.03	**.82**	.05
1. In a group, I often take the role of a leader.	.15	-.07	.01	**.82**	.00
5. Other group members turn to me for guidance.	.15	.24	-.03	**.66**	.13
4. I often do most of the strategic planning in the group I belong to.	.15	.18	.02	**.65**	.05
8. I take a central position in the group I belong to.	.12	.11	-.01	**.63**	-.11
6. Other group members usually do as I say.	.14	.12	.03	**.62**	-.09
12. I often "blindly" follow my group.	-.06	.05	-.17	-.18	**-.67**
16. I am willing to change my beliefs for the group I belong to.	.11	.02	.07	-.01	**-.63**
14. I am willing to do whatever it takes to get my group to accept me.	.06	.12	.02	.17	**-.59**
33. I care little about people outside my group.	.11	-.24	-.25	.01	**-.52**
17. I put the group first and myself second.	.12	.23	.22	-.01	**-.45**
41. Before I became part of my group, I had no clear direction in life.	.14	.09	-.23	-.14	**-.45**
Eigenvalues	16.15	4.84	3.98	2.17	1.69
% of variance explained	31.67	9.49	7.80	4.25	3.31
Cronbach’s alpha	.93	.74	.78	.91	.78

*Note*. Factor loadings > .40 are boldface. Traveler = Fellow Traveler

**Table 4:** Hierarchical Multiple Regression Analyses Predicting Violent Behavioral Intentions.

Predictor	∆*R*^*2*^	b (*SE*)	*β*	*p*	95% CI	
					LL	UL
Step 1	.24***					
Social Dominance Orientation		.14 (.06)	.16	.017	.025	.245
Right-Wing Authoritarianism		-.04 (.07)	-.04	.552	-.186	.100
Nationalism		.15 (.08)	.15	.055	-.003	.296
Ethnic Intolerance		.37 (.07)	.15	<.001	.234	.499
Ethnic Identification		-.10 (.06)	-.09	.122	-.220	.026
Step 2	.34***					
Social Dominance Orientation		.15 (.05)	.17	.007	.041	.253
Right-Wing Authoritarianism		.02 (.07)	.02	.771	-.116	.156
Nationalism		.03 (.08)	.03	.734	-.122	.173
Ethnic Intolerance		.26 (.07)	.26	<.001	.126	.389
Ethnic Identification		-.04 (.06)	-.04	.536	-.166	.087
Adventurer		.34 (.06)	.36	<.001	.211	.464
Fellow Traveler		-.04 (.08)	-.03	.599	-.204	.118
Leader		-.06 (.06)	-.06	.335	-.182	.062
Drifter		.13 (.07)	.11	.047	.002	.265
Misfit		-.03 (.08)	-.03	.676	-.196	.127

Note.

***p < .001

**Table 6:** Correlation between the Scales.

Scale	1.	2.	3.	4.	5.	6.	7.	8.	9.	10.	11.	12.	13.	14.	15.
1. Adventurer															
2. Fellow Traveler	.17**														
3. Leader	.63***	.20***													
4. Drifter	.22***	.09	.07												
5. Misfit	.35***	.41***	.19***	.31***											
6. Honesty-humility	-.25***	-.19**	-.20***	-.32***	-.35***										
7. Emotionality	-.43***	.22***	-.36***	.01	.02	-.03									
8. Extraversion	.37***	.03	.59***	-.25***	-.09	.01	-.31***								
9. Agreeableness	-.03	-.03	.03	-.28***	-.14*	.31***	-.12*	.34***							
10. Conscientiousness	-.20***	-.01	.10	-.41***	-.41***	.31***	-.10	.28***	.15**						
11. Openness to Experience	.15***	-.05	.22***	-.05	-.28***	.12*	-.13*	.27***	.14*	.31***					
12. Machiavellianism	.28***	.19**	.22***	.44***	.45***	-.67***	.00	-.06	-.32***	-.35***	-.05				
13. Psychopathy	.24***	.05	.10	.45***	.37***	-.43***	-.18**	-.26***	-.53***	-.33***	-.14*	.62***			
14. Narcissism	.38***	.31***	.38***	.24***	.43***	-.52***	-.02	.20***	-.24***	-.16**	-.04	.60***	.40***		
15. Violent Behavioral Intentions	.37***	-.02	.31***	.22***	.30***	-.18**	-.23***	.12*	-.16**	-.22***	-.04	.23***	.29***	.29***	
16. Radicalism Intentions	.34***	.13*	.20***	.26***	.35***	-.21***	-.08	-.01	-.12*	-.29***	-.02	.28***	.28***	.31***	.56***

*Note*.

*p < .05,

**p < .01,

***p < .001

**Table 7:** Psychometric Properties and Partial Correlations of the Scales.

Variable	Items	*M*	*SD*	Skew	Adventurer		Traveler		Leader		Drifter		Misfit	
					*r*_p_	*p*	*r*_p_	*p*	*r*_p_	*p*	*r*_p_	*p*	*r*_p_	*p*
Extremist Archetypes														
Adventurer	6	3.39	1.41	.23										
Fellow Traveler	6	4.43	0.90	-.73	-.06	.300								
Leader	6	3.83	1.35	-.06	.61	<.001	.15	.012						
Drifter	6	3.16	1.11	.47	.16	.006	-.04	.539	-.09	.136				
Misfit	6	2.63	0.96	.79	.25	<.001	.38	<.001	-.06	.274	.25	<.001		
Personality														
Honesty-humility	10	3.54	.72	-.17	-.04	.465	-.05	.409	-.08	.168	-.23	<.001	-.20	.001
Emotionality	10	3.15	.78	-.10	-.36	<.001	.30	<.001	-.13	.028	.07	.253	.09	.144
Extraversion	10	3.22	.80	-.20	.16	.005	-.03	.651	.51	<.001	-.33	<.001	-.19	.001
Agreeableness	10	3.31	.68	-.09	.04	.550	.01	.890	.03	.555	-.27	<.001	-.06	.349
Conscientiousness	10	3.85	.63	-.23	-.21	<.001	.17	.003	.29	<.001	-.28	<.001	-.39	<.001
Openness to Experience	10	3.66	.74	-.50	.12	.034	.07	.260	.16	.006	.05	.403	-.39	<.001
Dark Triad														
Machiavellianism	4	2.66	1.31	.71	.02	.789	-.001	.981	.10	.074	.35	<.001	.30	<.001
Psychopathy	4	2.56	1.16	.69	.07	.247	-.11	.050	-.02	.706	.37	<.001	.25	<.001
Narcissism	4	2.81	1.33	.57	.09	.146	.13	.027	.21	<.001	.16	.007	.24	<.001
Violence														
Violent Behavioral Intentions	7	2.58	1.35	.69	.14	.013	-.20	<.001	.15	.010	.14	.019	.20	.001
Radicalism Intentions	4	2.42	1.31	.65	.17	.003	-.01	.817	-.00	.950	.15	.012	.19	.001

*Note*. Potential range for the personality scales are 1–5; the rest range from 1–7. *r*_*p*_ = partial correlations. Control variables in the partial correlations are the remaining archetypes. Traveler = Fellow Traveler.

**Table 10:** Correlation between the Scales.

Scale	1	2	3	4	5	6	7	8	9	10	11	12	13	14	15	16	17
1. Adventurer																	
2. Fellow Traveler	.38**																
3. Leader	.71***	.33***															
4. Drifter	.22***	.31***	.26***														
5. Misfit	.29***	.51***	.27***	.35***													
6. Honesty-humility	-.22***	-.29***	-.30***	-.36***	-.31***												
7. Emotionality	-.24***	0.05	-0.11	-0.05	-0.01	-0.01											
8. Extraversion	.39***	-0.05	.48***	-.13*	-0.07	-0.02	-.15**										
9. Agreeableness	-0.02	-0.03	0.06	-.29***	-0.06	.32***	-0.04	.18**									
10. Conscientiousness	-0.1	-.20***	0	-.31***	-.33***	.37***	0.03	.20***	.20**								
11. Openness	.17**	-0.05	.21***	0.06	-.23***	-0.01	0	.24***	.15*	.24***							
12. Machiavellianism	.22***	.23***	.25***	.32***	.36***	-.60***	-0.05	-0.03	-.26***	-.43***	-0.09						
13. Psychopathy	.15**	.26***	.16**	.38***	.38***	-.47***	-.22***	-.19**	-.38***	-.39***	-.24***	.62***					
14. Narcissism	.21***	.28***	.30***	.29***	.30***	-.54***	0.07	0.08	-.21***	-.25***	0.01	.54***	.47***				
15. Violent Behavioral Intentions ethnic	.22***	.15**	.12*	.13*	.21***	-.29***	-.26***	-0.04	-.11*	-.24***	-0.09	.31***	.34***	.15**			
16. Violent Behavioral Intentions political	.22***	.19**	.16**	.16**	.28***	-.32***	-.24***	-0.1	-0.08	-.33***	-.16**	.34***	.40***	.19**	.74***		
17. Radicalism Intentions ethnic	.31***	.28***	.20***	.13*	.26***	-.30***	-.15**	-0.02	-.15**	-.26***	-0.04	.24***	.24***	0.11	.60***	.58***	
18. Radicalism Intentions political	.27***	.29***	.19**	.19**	.29***	-.33***	-.14*	-0.06	-.13*	-.29***	-0.06	.27***	.31***	.12*	.62***	.61***	.84***

Note.

*p < .05,

**p < .01,

***p <.001

**Table 11:** Psychometric Properties and Partial Correlations of the Scales.

Variable	Items	*M*	*SD*	Skew	Partial correlations									
					Adventurer		Traveler		Leader		Drifter		Misfit	
					*r*_p_	*p*	*r*_p_	*p*	*r*_p_	*p*	*r*_p_	*p*	*r*_p_	*p*
Extremist Archetypes														
Adventurer	6	3.93	1.23	-.24										
Fellow Traveler	6	4.21	0.88	-.39	.18	<.001								
Leader	6	3.82	1.14	.13	.66	<.001	.03	.579						
Drifter	6	3.31	0.98	.08	-.02	.734	.13	.027	.13	.023				
Misfit	6	2.66	0.91	.35	.05	.429	.41	<.001	.04	.497	.22	<.001		
Personality														
Honesty-humility	10	3.56	.65	-.36	.05	.371	-.09	.121	-.17	.003	-.24	<.001	-.12	.028
Emotionality	10	3.35	.61	-.35	-.26	<.001	.15	.009	.09	.122	-.05	.374	-.01	.904
Extraversion	10	3.17	.63	-.24	.16	.005	-.16	.005	.38	<.001	-.23	<.001	-.11	.059
Agreeableness	10	3.29	.59	-.12	.04	.514	.04	.461	-.03	.602	-.29	<.001	-.02	.730
Conscientiousness	10	3.57	.56	-.16	-.09	.122	-.02	.726	.17	.003	-.24	<.001	-.23	<.001
Openness to Experience	10	3.39	.64	-.12	.08	.179	-.01	.884	.16	.005	.11	.061	-.30	<.001
Dark Triad														
Machiavellianism	4	2.32	1.18	.93	.02	.768	-.00	.949	.08	.121	.12	.001	.23	<.001
Psychopathy	4	2.41	1.05	.82	.00	.980	.03	.555	.0	.997	.28	<.001	.24	<.001
Narcissism	4	3.02	1.37	.32	-.05	.366	.10	.083	.18	.002	.15	.007	.13	.018
Violence														
Violent Behavioral Intentions ethnic	7	2.28	1.27	.79	.12	.035	-.00	.973	-.07	.209	.05	.384	.13	.027
Violent Behavioral Intentions political	7	2.12	1.17	.80	.17	.003	.02	.750	-.03	.614	.05	.368	.18	.001
Radicalism Intentions ethnic	4	2.71	1.51	.60	.20	< .001	.11	.043	-.05	.371	.01	.877	.12	.042
Radicalism Intentions political	4	2.53	1.40	.64	.14	.011	.12	.032	-.04	.532	.06	.257	.13	.018

Note. Potential range for the personality scales are 1–5; the rest range from 1–7. r_p_ = partial correlations. Control variables in the partial correlations are the remaining archetypes. Traveler = Fellow Traveler.

**Table 12:** Psychometric Properties and Partial Correlations Between the Extremist Archetypes and the HEXACO Facets.

Variable	Items	α/*r* ^a^	*M*	*SD*	Skew	Partial correlations									
						Adventurer		Traveler		Leader		Drifter		Misfit	
						*r*_p_	*p*	*r*_p_	*p*	*r*_p_	*p*	*r*_p_	*p*	*r*_p_	*p*
Honesty-humility															
Sincerity	3	.60	3.55	0.86	-.29	.08	.153	-.09	.107	-.06	.322	**-.16**	**.005**	-.07	.214
Fairness	3	.73	3.37	0.39	-1.15	.01	.875	-.09	.120	-.07	.226	**-.14**	**.016**	**-.16**	**.006**
Greed-Avoidance	2	.43*	2.96	0.98	-.01	.06	.305	-.10	.092	**-.15**	**.007**	**-.17**	**.003**	.06	.272
Modesty	2	.41*	3.69	0.87	-.33	-.03	.656	.09	.102	**-.24**	**<.001**	**-.12**	**>.010**	**-.15**	**.010**
Emotionality															
Fearfulness	3	.55	3.25	0.79	-.14	**-.29**	**<.001**	**.14**	**.015**	.06	.332	-.08	.172	.04	.537
Anxiety	2	.44*	3.67	0.85	-.30	**-.26**	**<.001**	**.19**	**.001**	.08	.150	.05	.390	-.0	.252
Dependence	2	.36*	2.88	0.92	-.21	**-.17**	**.003**	.06	.255	.09	.102	.06	.278	.03	.614
Sentimentality	3	.62	3.55	0.78	-.47	-.07	.209	.06	.298	.04	.499	**-.13**	**.018**	-.03	.623
Extraversion															
Social Self-Esteem	3	.66	3.32	0.85	-.32	.06	.311	-.09	.126	**.29**	**<.001**	**-.25**	**<.001**	**-.15**	**.009**
Social Boldness	3	.66	2.96	0.80	-.12	**.13**	**.027**	**-.16**	**.005**	**.38**	**<.001**	-.07	.254	-.07	.247
Sociability	2	.36*	3.14	0.92	-.27	**.17**	**.002**	**-.15**	**.010**	**.18**	**.001**	-.06	.273	.06	.317
Liveliness	2	.36*	3.26	0.78	-.25	.11	.058	-.05	.372	**.22**	**<.001**	**-.26**	**<.001**	**-.12**	**.032**
Agreeableness															
Forgiveness	2	.67*	3.17	1.04	-.20	.07	.238	-.02	.728	-.01	.826	**-.22**	**<.001**	**.12**	**.031**
Gentleness	3	.45	3.38	0.68	-.22	**.13**	**.024**	.04	.484	-.08	.161	**-.19**	**.001**	-.03	.598
Flexibility	3	.34	3.18	0.68	.03	-.05	.405	.03	.633	.01	.852	**-.18**	**.002**	.01	.891
Patience	2	.57*	3.43	0.95	-.29	-.05	.399	.08	.167	.00	.957	**-.26**	**<.001**	-.05	.358
Conscientiousness															
Organization	2	.26*	3.70	0.81	-.29	-.07	.216	-.03	.561	**.13**	**.028**	**-.19**	**.001**	**-.14**	**.012**
Diligence	2	.33*	3.77	0.77	-.64	.10	.080	-.05	.416	**.14**	**.016**	-.11	.063	**-.25**	**<.001**
Perfectionism	3	.48	3.56	0.70	-.27	-.03	.652	.04	.442	**.15**	**.007**	**-.17**	**.003**	**-.12**	**.031**
Prudence	3	.68	3.38	0.79	-.19	**-.20**	**<.001**	-.03	.541	.07	.211	**-.21**	**<.001**	**-.17**	**.003**
Openness to Experience															
Aesthetic Appreciation	2	.45*	3.14	1.12	-.19	-.02	.759	-.03	.658	**.15**	**.010**	.11	.062	**-.20**	**<.001**
Inquisitiveness	2	.25*	3.66	0.85	-.34	-.02	.714	-.01	.907	**.17**	**.004**	.02	.699	**-.27**	**<.001**
Creativity	3	.67	3.43	0.91	-.22	.09	.086	-.06	.326	**.13**	**.024**	**.15**	**.010**	**-.19**	**.001**
Unconventionality	3	.35	3.33	0.70	.21	**.13**	**.018**	.08	.157	.01	.815	-.01	.934	**-.22**	**<.001**

Note. r_p_ = partial correlations. Control variables in the partial correlations are the remaining four archetypes. p < .05 are in boldface.

^a^Alpha coefficients are presented for scales with more than two items. Correlation coefficients are presented for two items, *p <.001.

Traveler = Fellow Traveler.
