# Supplementary material for: Measuring extremist archetypes: Scale development and validation
Source: PLoS One. 2022 Jul 20;17(7):e0270225. doi: 10.1371/journal.pone.0270225 (PMC9299337; doi:10.1371/journal.pone.0270225)
Supplement: S1 File — (DOCX) [file pone.0270225.s001.docx]

**Supplemental Online Material**

**Table S2**

*Sample characteristics for Study 3 (N =303 )*

| Characteristic |  | % |
| --- | --- | --- |
| **Gender** | Male  Female  Other | 33.4  65.6  .9 |
| **Age** | 18-29  30-49  50-76 | 56.4  54.27  2.13 |
| **Completed education** | Less than high school degree  High school  Some college  Associate degree in college (2)  Bachelor degree (4 year)  Master’s degree  Doctoral degree  Professional degree | .6  13.6  15.1  9.5  37.5  19.6  2.2  1.9 |
| **Income** | Less than £15,000  £15,000 to £24,999  £25,000 to £34,999  £35,000 to £49,999  £50,000 to £74,999  £75,000 to £99,999  £100,000 to £149,999  £150,000 or more |  |
| **Criminal activity** | No previous activity.  Previous (nonviolent) minor activity  Previous (nonviolent serious activity  Previous violent crime | .9  -  -  - |

**Study 1**

All 51 items and the corresponding factor loadings from the exploratory factor analysis with direct oblimin rotation.

**Measures**

| **Final 30 items of the Extremist Archetypes Scale.** |  |
| --- | --- |
| The scale is introduced with the following text; Most people have some opinions regarding political issues and often support specific political movements. We would like you to think of the political group or movement you belong to or feel close to when answering the following questions. Please indicate to which extent you agree with the various statements regarding yourself. |  |
| Adventurer |  |
| 1. I am known as a group member that is not afraid to take any risks. |  |
| 2. I am known as a group member that is not afraid of facing dangers. |  |
| 3. I am willing to take more risks than other people in my group. |  |
| 4. Other group members see me as someone who likes thrills and adventure. |  |
| 5. I like groups that bring along adventure and adrenaline. |  |
| 6. I like to show other group members that I am not afraid of taking risks. |  |
| Fellow Traveler |  |
| 7. I try to find a group that accepts me. |  |
| 8. To belong to a group, I am willing to do what is asked of me. |  |
| 9. I tend to "go with the flow" in group settings. |  |
| 10. The group I belong to gives me stability in life. |  |
| 11. I often join groups that others have introduced me to. |  |
| 12. I stay in a group as long as they give me what I need. |  |
| Leader |  |
| 13. Most group members see me as a leader. |  |
| 14. In a group, I often take the role of a leader. |  |
| 15. Other group members turn to me for guidance. |  |
| 16. I often do must of the strategic planning in the group I belong to. |  |
| 17. I take a central position in the group I belong to. |  |
| 18. Other group members usually do as I say. |  |
| Drifter |  |
| 19. Sometimes, I suddenly decide to leave a group and seek a new one. |  |
| 20. Throughout my life I have found it difficult to find a group to belong to. |  |
| 21. It has happened that I just replaced one group I belonged to with another. |  |
| 22. I rarely stay with one group for a longer period of time. |  |
| 23. I have shifted a lot between groups in my life. |  |
| 24. I get bored more easily than other group members. |  |
| Misfit |  |
| 25. I often "blindly" follow my group. |  |
| 26. I am willing to change my beliefs for the group I belong to. |  |
| 27. I am willing to do whatever it takes to get my group to accept me. |  |
| 28. I care little about people outside my group. |  |
| 29. I put the group first and myself second. |  |
| 30. Before I became part of my group, I had no clear direction in life. | |
| *Note*. Distribute in a randomized order. Responses are rated on a 7-point Likert scale ranging from 1 ("strongly disagree"), to 4 ("neither agree nor disagree"), to 7 ("strongly agree"). | |

**8-item SDO7 (Ho et al., 2015**

- We should work to give all groups an equal chance to succeed.
- An ideal society requires some groups to be on top and others to be on the bottom.
- Some groups of people are simply inferior to other groups.
- No one group should dominate in society.
- Groups at the bottom are just as deserving as groups at the top.
- Group equality should not be our primary goal.
- It is unjust to try to make groups equal.
- We should do what we can to equalize conditions for different groups.

**Nationalism (Weiss, 2003)**

- One should only help other countries if this is to the advantage of one's own country.
- It is the foremost duty of each young American to honor the national history and its heritage.
- Because of our important historical experience, we should have more to say in international affairs.
- If other countries accepted more of what we do here, they would be better off.

**Violent Intention (Obaidi et al., 2018)**

- As a last resort I'm personally ready to use violence for the sake of my ethnic group.
- If nothing else helps, I'm prepared to use violence to defend my ethnic group.
- I'm ready to go and fight for my ethnic group in another country.
- I will not personally use violence to help my ethnic group.
- I'm not prepared to use violence in any situation.
- I will personally use violence against people harming other ethnic group members that I care about.
- Even as a last resort, I will not use violence for the sake of other ethnic group members.

**Right-Wing Authoritarianism Scale (Zakarisson, 2005)**

- Our country needs a powerful leader, in order to destroy the radical and immoral currents prevailing in society today.
- Our country needs free thinkers, who will have the courage to stand up against traditional ways, even if this upsets many people.
- The ?old-fashioned ways? and ?old-fashioned values? still show the best way to live.
- Our society would be better off if we showed tolerance and understanding for untraditional values and opinions.
- God?s laws about abortion, pornography and marriage must be strictly followed before it is too late, violations must be punished.
- The society needs to show openness towards people thinking differently, rather than a strong leader, the world is not particularly evil or dangerous.
- It would be best if newspapers were censored so that people would not be able to get hold of destructive and disgusting material.
- Many good people challenge the state, criticize the church and ignore ?the normal way of living?.
- Our forefathers ought to be honored more for the way they have built our society, at the same time we ought to put an end to those forces destroying it.
- People ought to put less attention to the Bible and religion, instead they ought to develop their own moral standards.
- There are many radical, immoral people trying to ruin things; the society ought to stop them.
- It is better to accept bad literature than to censor it.
- Facts show that we have to be harder against crime and sexual immorality, in order to uphold law and order.
- The situation in the society of today would be improved if troublemakers were treated with reason and humanity.
- If the society so wants, it is the duty of every true citizen to help eliminate the evil that poisons our country from within.

**Ethnic Intolerance Scale (Weiss, 2003)**

- If there are too many foreigners in the country, one might as well let them feel that they are not welcome.
- It is better if only members of the same people get married to each other.
- It would be best if every people also had its own state.
- You can only feel secure if your own people is in the majority.

**Self-Categorization Scale (Ellemers et al., 1999)**

- My ethnic group is an important reflection of who I am.
- I identify with other members of my ethnic group.
- I am like other members of my ethnic group.

**HEXACO-60 (Ashton & Lee, 2009)**

*Openness*

- I would be quite bored by a visit to an art gallery.
- I think that paying attention to radical ideas is a waste of time.
- I find it boring to discuss philosophy.
- I’ve never really enjoyed looking through an encyclopedia.
- I don’t think of myself as the artistic or creative type.
- I'm interested in learning about the history and politics of other countries.
- I like people who have unconventional views.
- I would enjoy creating a work of art, such as a novel, a song, or a painting.
- If I had the opportunity, I would like to attend a classical music concert.
- People have often told me that I have a good imagination.

*Emotionality*

- I can handle difficult situations without needing emotional support from anyone else.
- I worry a lot less than most people do.
- Even in an emergency I wouldn’t feel like panicking.
- I remain unemotional even in situations where most people get very sentimental
- I would feel afraid if I had to travel in bad weather conditions.
- When I suffer from a painful experience, I need someone to make me feel comfortable.
- When it comes to physical danger, I am very fearful.
- I feel strong emotions when someone close to me is going away for a long time.
- I sometimes can't help worrying about little things.
- I feel like crying when I see other people crying.

*Extraversion*

- I rarely express my opinions in group meetings.
- Most people are more upbeat and dynamic than I generally am.
- I sometimes feel that I am a worthless person.
- I feel that I am an unpopular person.
- I feel reasonably satisfied with myself overall.
- I prefer jobs that involve active social interaction to those that involve working alone.
- On most days, I feel cheerful and optimistic.
- In social situations, I’m usually the one who makes the first move.
- When I’m in a group of people, I’m often the one who speaks on behalf of the group.
- The first thing that I always do in a new place is to make friends.

*Consciousness*

- I make decisions based on the feeling of the moment rather than on careful thought.
- When working on something, I don't pay much attention to small details.
- When working, I sometimes have difficulties due to being disorganized.
- I do only the minimum amount of work needed to get by.
- I make a lot of mistakes because I don’t think before I act.
- I prefer to do whatever comes to mind, rather than stick to a plan.
- I plan ahead and organize things, to avoid scrambling at the last minute.
- I often push myself very hard when trying to achieve a goal.
- I always try to be accurate in my work, even at the expense of time.
- People often call me a perfectionist.

*Honesty-humility*

- If I want something from someone, I will laugh at that person's worst jokes.
- If I knew that I could never get caught, I would be willing to steal a million dollars.
- I think that I am entitled to more respect than the average person is.
- I would get a lot of pleasure from owning expensive luxury goods.
- I want people to know that I am an important person of high status.
- I’d be tempted to use counterfeit money, if I were sure I could get away with it.
- I wouldn't use flattery to get a raise or promotion at work, even if I thought it would succeed.
- Having a lot of money is not especially important to me.
- I would never accept a bribe, even if it were very large.
- I wouldn’t pretend to like someone just to get that person to do favors for me.

*Agreeableness*

- People sometimes tell me that I am too critical of others.
- People sometimes tell me that I'm too stubborn.
- People think of me as someone who has a quick temper.
- When people tell me that I’m wrong, my first reaction is to argue with them.
- I rarely hold a grudge, even against people who have badly wronged me.
- My attitude toward people who have treated me badly is “forgive and forget”.
- I tend to be lenient in judging other people.
- I am usually quite flexible in my opinions when people disagree with me.
- Most people tend to get angry more quickly than I do.
- Even when people make a lot of mistakes, I rarely say anything negative.

**The Dirty Dozen Scale (Jonason & Webster, 2010)**

- I tend to manipulate others to get my way.
- I have used deceit or lied to get my way.
- I have used flattery to get my way.
- I tend to exploit others towards my own end.
- I tend to lack remorse.
- I tend to be unconcerned with the morality of my actions.
- I tend to be callous or insensitive.
- I tend to be cynical.
- I tend to want others to admire me.
- I tend to want others to pay attention to me.
- I tend to seek prestige or status.
- I tend to expect special favors from others.

**Radicalism Intention Scale (Moskalenko & McCauley, 2009)**

- I would attack police or security forces if I saw them beating members of my ethnic group.
- I would continue to support a group that fights for my ethnic group's political and legal rights even if the group sometimes breaks the law.
- I would continue to support a group that fight's for my ethnic group's political and legal rights even if the groups sometimes resorts to violence.
- I would participate in a public protest against oppression of my ethnic group even if I thought the protest might turn violent.
